# Single-cell transcriptomic analysis reveals the developmental trajectory and transcriptional regulatory networks of *quinoa* salt bladders

**DOI:** 10.1007/s44154-024-00189-3

**Published:** 2024-11-13

**Authors:** Hao Liu, Zhixin Liu, Yaping Zhou, Aizhi Qin, Chunyang Li, Yumeng Liu, Peibo Gao, Qianli Zhao, Xiao Song, Mengfan Li, Luyao Kong, Yajie Xie, Lulu Yan, Enzhi Guo, Xuwu Sun

**Affiliations:** National Key Laboratory of Cotton Bio-Breeding and Integrated Utilization, State Key Laboratory of Crop Stress Adaptation and Improvement, School of Life Sciences, Henan University, Kaifeng, 475001 China

**Keywords:** *Quinoa*, Salt bladders, Single-cell RNA sequencing, Developmental trajectory, Salt tolerance

## Abstract

Salt bladders, specialized structures on the surface of *quinoa* leaves, secrete Na^+^ to mitigate the effects of the plant from abiotic stresses, particularly salt exposure. Understanding the development of these structures is crucial for elucidating *quinoa*’s salt tolerance mechanisms. In this study, we employed transmission electron microscopy to detail cellular differentiation across the developmental stages of *quinoa* salt bladders. To further explore the developmental trajectory and underlying molecular mechanisms, we conducted single-cell RNA sequencing on *quinoa* protoplasts derived from young leaves. This allowed us to construct a cellular atlas, identifying 13 distinct cell clusters. Through pseudotime analysis, we mapped the developmental pathways of salt bladders and identified regulatory factors involved in cell fate decisions. GO and KEGG enrichment analyses, as well as experimental results, revealed the impacts of salt stress and the deprivation of sulfur and nitrogen on the development of *quinoa* salt bladders. Analysis of the transcription factor interaction network in pre-stalk cells (pre-SC), stalk cells (SC), and epidermal bladder cells (EBCs) indicated that *TCP5*, *YAB5*, *NAC078*, *SCL8*, *GT-3B*, and *T1P17.40* play crucial roles in EBC development. Based on our findings, we developed an informative model elucidating salt bladder formation. This study provides a vital resource for mapping *quinoa* leaf cells and contributes to our understanding of its salt tolerance mechanisms.

**Supplementary Information:**

The online version contains supplementary material available at 10.1007/s44154-024-00189-3.

## Introduction

Soil salinity has a serious impact on crop productivity, and improving salt tolerance has become an important strategy for genetic breeding to maintain the world’s food supply, improve land use efficiency, and maintain crop yields (Elhindi et al. 2017). *Quinoa* (*Chenopodium quinoa* Willd.) is an annual herb that can grow under adverse abiotic stresses such as high salinity, drought, and cold (Jacobsen et al. 2003). Originally from the Andean region of South America, *quinoa* is a food crop with high nutritional and commercial value (Angeli et al. 2020). *Quinoa* contains a large number of phytochemicals with health benefits, including amino acids, fibers, polyunsaturated fatty acids, vitamins, minerals, saponins, phytosterols, phytoalexins, phenols, betaine, and glycine betaine (Graf et al. 2015). In addition, *quinoa* is gluten-free, making it particularly suitable for people with wheat allergies or intolerances (Bian et al. 2023). *Quinoa* is a dicotyledonous C3 salt plant, but it is often mistaken for a cereal such as maize and wheat (monocotyledons of the grass family), hence the term ‘pseudo-cereal’ (Graf et al. 2015). Similar to most leaves, the adaxial and abaxial leaf surfaces of *quinoa* contain many epidermal bladder cells (EBCs), the density of which varies significantly with leaf age, with the highest density in young leaves (L. Shabala et al. 2012). Epidermal bladder cells are modified trichomes, spherical in shape, and each EBC is ten times larger in diameter than an epidermal cell (EC), thus able to absorb up to 1,000 times more Na^+^ than an EC (Shabala et al. 2014). Studies suggest that *quinoa* salt bladders protect plant cells from UV damage and that their accumulated organic osmoregulators scavenge reactive oxygen species and reduce water loss (Adolf et al. 2013; Shabala et al. 2012).

The EBCs on the surface of *quinoa* leaves form the EC-SC-EBC complex together with the stalk cell (SC) and the epidermal cell (EC) at the base (Shabala et al. 2014). SCs contain most organelles such as mitochondria, chloroplasts, endoplasmic reticulum, Golgi apparatus, and plasmodesmata often appear in close contact to vesicular structures (Bazihizina et al. 2022). By analogy with the pattern of epidermal cells and trichome formation in *Arabidopsis*, a simplified model of salt bladder organogenesis was proposed: during vesicle complex formation, the ‘doomed’ epidermal cell extends slightly to divide into two cells, giving rise to a lower and upper segment. The upper segment then extends outwards and forms the trichome initial, which divides again to form the apical and basal cells. The apical cell increases in size and develops a large central vesicle, which eventually turns into a spherical vesicle, while the basal cell expands a little and develops into a stalk cell (Shabala et al. 2014). In a recent study, Zhang et al. studied the developmental process of salt bladders by paraffin sectioning of Chenopodium album leaves at different developmental stages, and the results were generally consistent with the simplified model of salt bladder histogenesis described above (Zhang et al. 2022).

To regulate the osmotic potential balance between the cytoplasm and the vesicles and to maintain the normal function of the organelles, plants accumulate soluble substances in the cytoplasm that do not affect the enzymatic activity and the structure and function of biomolecules to reduce the osmotic potential in the cytoplasm. The main osmolytes reported to be involved in osmoregulation in *quinoa* include proline, inositol, and glycine betaine (Jacobsen et al. 2007; Kiani‐Pouya et al. 2017; Ruffino et al. 2009). Proline is an osmotic agent known to be involved in plant salt responses, which can mitigate the adverse effects of stressful environments in a variety of ways, including protecting cell structure, protein integrity, and enhancing enzyme activity (Szabados & Savouré 2010). Inositol also plays an important role in plant osmoregulation, and inositol transport may be closely coupled to Na^+^ transport (Adams et al. 2005). In addition to its direct involvement in the regulation and protection of vesicle-like membranes, glycine betaine may also indirectly protect cells from environmental stress through its action in signal transduction (Ashraf & Foolad 2007). A metabolic study of EBCs from *quinoa* under various abiotic stresses revealed that some primary metabolites showed significant metabolic responses under heat, cold, and intense light stresses, with little change in secondary metabolites. Under salt and heat stress, the lipid composition in EBCs changed significantly (Otterbach et al. 2021). Using high-performance liquid chromatography, it was found that UV treatment induced a rapid accumulation of large amounts of beetroot heme, flavonoids, and other substances in salt bladders (Vogt et al. 1999). Betaine is a natural pigment in plants and it is believed that the pigment in plant epidermal cells absorbs UV light and reduces UV damage to plants (Wang & Wang 2007). Flavonoids are secondary metabolites synthesized by plants that can absorb UV light and resist peroxidation (Ichino et al. 2020). Salt bladders can store up to 1 M NaCl within a giant vesicle, and Na^+^ and Cl^−^ need to cross the plasma membrane (PM), traverse the cytoplasm, and be loaded into the vesicle lumen (Böhm et al. 2018). EBCs can improve salt tolerance through xylem Na^+^ loading, high reactive oxygen species (ROS) tolerance, K^+^ homeostasis, and effective control of stomatal development. EBCs can improve plant salt tolerance in a variety of ways, including through xylem Na^+^ loading, high reactive oxygen species (ROS) tolerance, K^+^ homeostasis, and effective control of stomatal development (Adolf et al. 2013). The plasma membrane ion transporter *SOS1* (*salt overly sensitive 1*) and *HKT1* (*high-affinity K*^+^ *transporters 1*) systems are the main players in plant Na^+^ transport across the PM (Shi et al. 2002; Waters et al. 2013). *SOS1* represents the Na^+^/H^+^-antiporter protein that uses proton-powered proton-motive-force (PMF) to move Na^+^ out of the cell (Qiu et al. 2002).

One study reported high expression of genes homologous to the vesicular membrane Na^+^/H^+^ reverse transporter protein *NHX* (Na^+^/H^+^ *exchanger*) in the transcriptome data of *quinoa* salt bladders (Zou et al. 2017). When salt bladders accumulate salt to a certain level, they will undergo autophagy, and apoptotic rupture, thus avoiding or mitigating the damage caused by stresses such as NaCl (Hu et al. 2020). The presence of large vesicles of EBCs implies vesicle development and transport of vesicle-associated proteins. Vesicular proteins fuse with the vesicle membrane, thereby entering the vesicle and participating in vesicle development (Hu et al. 2020). During this process, the *VPS4* (*vacuolar protein sorting 4*) gene is involved in endocytosis for transport to the vesicle and in the transport of vesicular proteins in the late Golgi phase (Finken-Eigen et al. 1997; Rothman & Stevens 1986). Echidna associated with green V-ATPase is a vesicular H^+^ pump that fuels *NHX* in the vesicular membrane, allowing Na^+^ transport into the vesicles, improving vesicular osmoregulation and maintaining Na^+^ and K^+^ homeostasis. Compared to other cell types, vesicular ATPase activity is constitutively high in the guard cell (GC) to meet the high and fast ion flux to the vesicular membrane required for stomatal movement (Bose et al. 2014; Rasouli et al. 2021). There are many GC proteins involved in resistance to salt stress, such as *auxin binding protein 19* (*ABP19*) that decreases GC cytoplasmic pH and induces stomatal opening; dehydrin *early responsive to dehydration* (*ERD14*) and *LOW-TEMPERATURE-INDUCED 65* (*LTI65*) attract water into the cell, regulate the osmotic potential and maintain water status (Rasouli et al. 2021).

Single-cell transcriptome sequencing technology has emerged as a powerful tool for plant research, allowing a deeper understanding of cellular heterogeneity during cell development and stress response. High-throughput single-cell transcriptome sequencing was performed on *Arabidopsis* root tissue protoplasts to resolve the developmental trajectories of root hair cells, and to confirm the feasibility and effectiveness of high-throughput single-cell sequencing in plants (Ryu et al. 2019). Regulators of epidermal development under drought and salt stress conditions were identified using single-cell RNA sequencing, highlighting the sensitivity of epidermal cells to environmental stress (Liu et al. 2022). By employing single-cell transcriptome sequencing, the developmental trajectory of pigment gland cells and the regulatory network of transcription factors were analyzed, leading to the proposal of a relatively complete model for the formation of pigment glands (Sun et al. 2023). By constructing a single cell resolution transcription map of cotton root tips in response to salt stress, the cell heterogeneity, root type difference and differentiation trajectory of plant roots under salt stress were revealed, which laid a solid foundation for elucidating the molecular mechanism of plant resistance (Li et al. 2024).

EBCs, as one of the important structures of salt tolerance in *quinoa*, are of great interest in studying the mechanism of tolerance to salt stress in *quinoa*. Genes related to membrane transporter proteins and those involved in the response to salt stress have been identified through RNA sequencing (RNA-seq) analysis of epidermal bladder cells (EBC) (Zou et al. 2017). In recent years, single-cell transcriptome sequencing technologies have significantly contributed to understanding the development of specific plant tissues and cell types, revealing new cell types, key regulatory genes for cell lineage developmental trajectories and fate determination, and molecular mechanisms of environmental adaptation. However, the molecular regulatory mechanisms underlying *quinoa* salt bladder development are currently unclear and studies at the high-throughput single-cell level are lacking. Therefore, in this study, we constructed a cellular map of young *quinoa* leaves using scRNA-seq. We classified the major cell types, screened for differentially expressed genes that are specifically expressed in different cell types, and identified key regulators. The developmental trajectories of the different cell types were studied by mimetic time series analysis. Using GO analysis of DEGs, we found that genes involved in the regulation of sulfur and nitrogen metabolism are highly expressed in EBCs. Further physiological and cell biological analyses showed that low sulfur (LS) treatment as salt treatment, promotes EBC development, whereas low nitrogen (LN) treatment inhibits EBC development. This suggests that genes involved in the regulation of sulfur and nitrogen metabolism could directly regulate EBC development and thus further regulate *quinoa* tolerance to salt stress. Our study provides a comprehensive resolution of the developmental processes of epidermal bladder cells at the single-cell level and sheds light on the pivotal role of soil sulfur and nitrogen in enhancing the growth and salt tolerance of *quinoa*. These findings offer novel insights into the molecular mechanisms underlying the development of salt bladders and establish a theoretical basis for the development of salt-tolerant crop varieties.

## Results

### Structural and developmental dynamics of salt bladders in *quinoa*

**Fig. 1:**
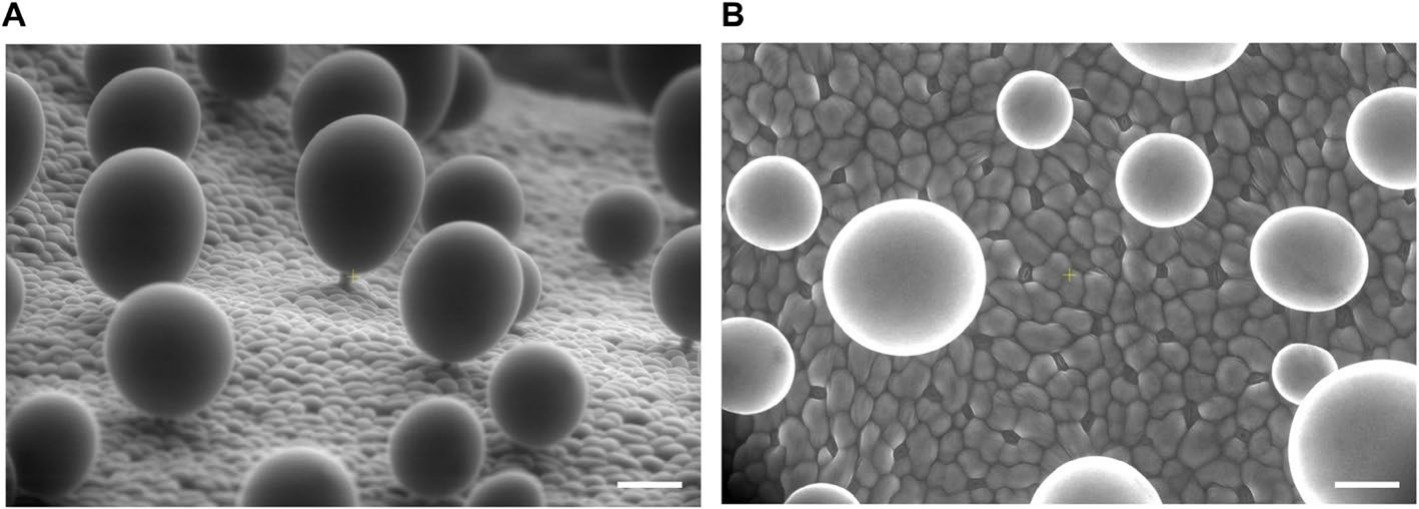
Surface morphology and structural characteristics of salt bladders observed by SEM. **A** Horizontal view of epidermal salt bladders on the leaf surface, showing their phenotype and distribution. **B** Vertical view of salt bladders on the upper epidermis of leaves, illustrating their phenotype and distribution. Scale bar: 50 μm

As a typical feature of salt-tolerant plants, the salt bladders on the leaf surface of *quinoa* are essential for regulating its ion balance and cellular metabolism. Scanning electron microscopy (SEM) of salt bladders on the upper epidermis of *quinoa* leaves grown under normal conditions for three weeks showed that mature salt bladders consist of a short stalk and gourd-shaped bladders that are narrower at the bottom and wider at the top (Fig. 1A and B). Mature salt bladders were much larger than epidermal cells, with diameters around 100 μm, and were scattered on the leaf surface (Fig. 1A-B). Figure 1C showed a schematic model of salt bladder development, with the undifferentiated initial bladder cells having divided by pericycle division to form pre-stalk cells and anterior epidermal bladder cells. Subsequent gradual expansion of the vesicles in the epidermal bladder cell pushes the major organelles to the cell edge. The pre-stalk cell differentiates or divides further perpendicularly to form a mature stalk cell that joins with the epidermal bladder cell above.

**Fig. 2:**
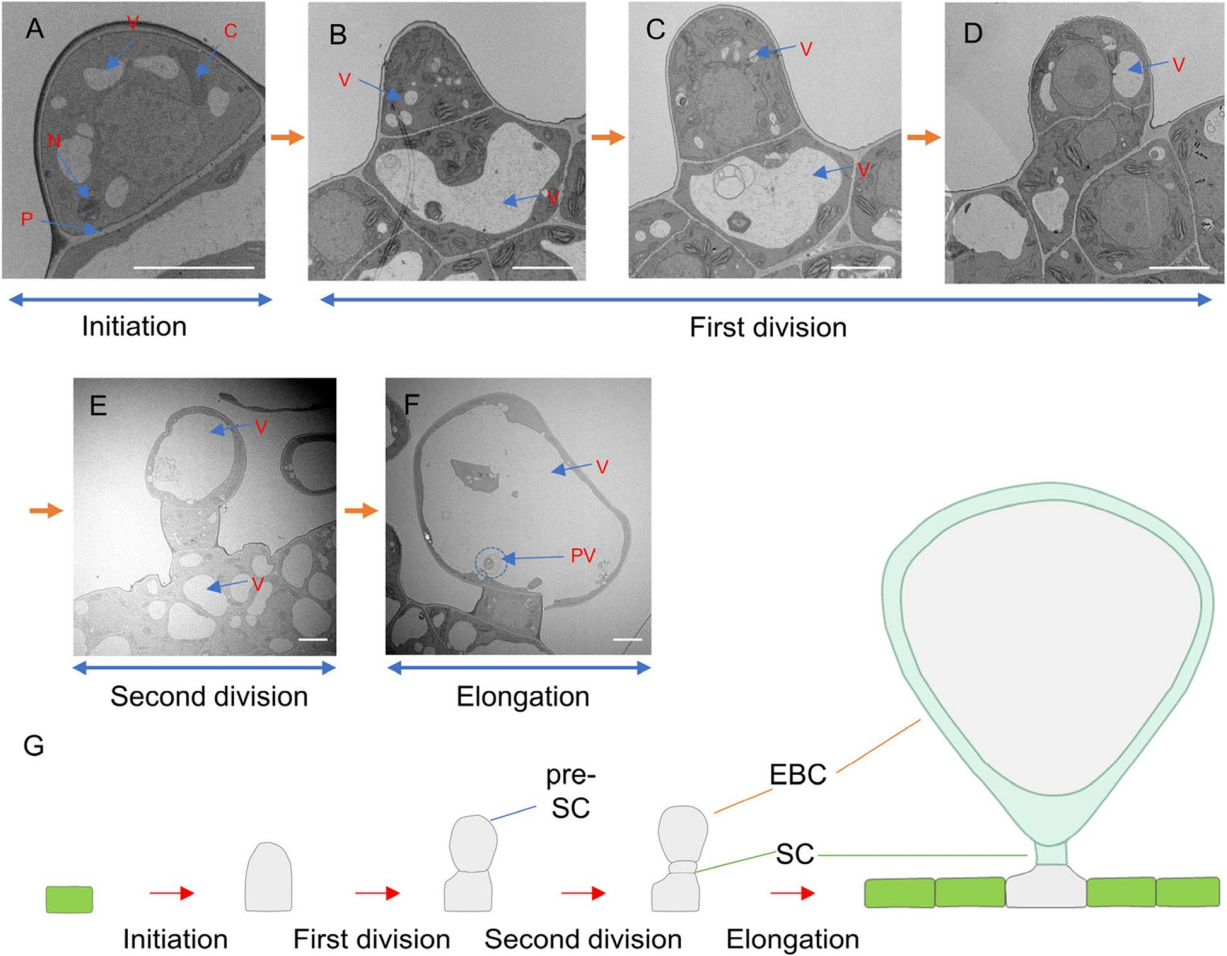
Developmental analysis of *quinoa* salt bladders using transmission electron microscopy. **A** Development of the initial bladder cell and the changes in internal organelles. **B**-**D** Division of the initial bladder cell into pre-stalk and epidermal cells. **E** Further division of pre-stalk cells into stalk cells and epidermal bladder cells. **F** Formation of mature salt bladders and the changes in internal organelles. **G** Model illustrating the formation and developmental progression of salt bladders. V: vacuole, N: nuclei, C: chloroplasts, P: plasmodesmata, PV: phagocytic vacuole. Scale bar: 5 μm

To observe the developmental process of the salt bladder in more detail, we used transmission electron microscopy (Rasouli, et al. 2021) on salt bladders on the true leaves of two-week-old *quinoa* seedlings. The results showed that the precursor cells divided and differentiated twice to form bladder cells (Fig. 2A-F). Furthermore, a model for the development of salt bladder was proposed (Fig. 2G). In the initial bladder cell that just protrudes, vesicles were dispersed (Fig. 2A). The epidermal cells adjacent to the initial bladder cells transformed through vesiculation, establishing a foundation for the absorption and provisional salt retention (Fig. 2B). Lysosomes within the epidermal cells encapsulate chloroplasts and senescent organelles, forming autophagic vesicles that digested and integrated their contents into the vacuolar compartment (Fig. 2B-C). During the development of bladder cells, vacuoles progressively enlarged, pushing chloroplasts, Golgi apparatuses, and endoplasmic reticulums to the cellular periphery, where they were subsequently engulfed and digested by the expanding vacuolar system (Fig. 2E-F).

We also analyzed further the ultrastructure of the initiating bladder cells, EBCs, and mature stalk cells (Fig. S1-S3). Initial bladder cells contained nuclei, Golgi apparatus, ribosomes, mitochondria, and chloroplasts. The small vesicles were not aggregated into large vesicles, and there were also a large number of plasmodesmata between initial bladder cells and neighboring epidermal cells for the transport of insoluble substances into the vesicles (Fig. S1). The cytoplasm of mature epidermal cells and salt bladder cells was relatively dense. The salt bladder consisted of EBCs and stalk cells, with the vesicles in the EBCs occupying the vast majority of the space, squeezing the organelles to the edges of the large vesicles (Fig. S2). Chloroplasts, mitochondria, Golgi apparatus, and endoplasmic reticulum can be seen in mature stalk cells. Among them, chloroplasts gradually shrank and were subsequently endocytosed and digested by the vesicles. There were a large number of mitochondria and Golgi bodies in the mature stalk cells, which may provide energy for cellular activities (Fig. S3).

### Construction of a single cell transcription atlas of young true leaves of *quinoa*

**Fig. 3:**
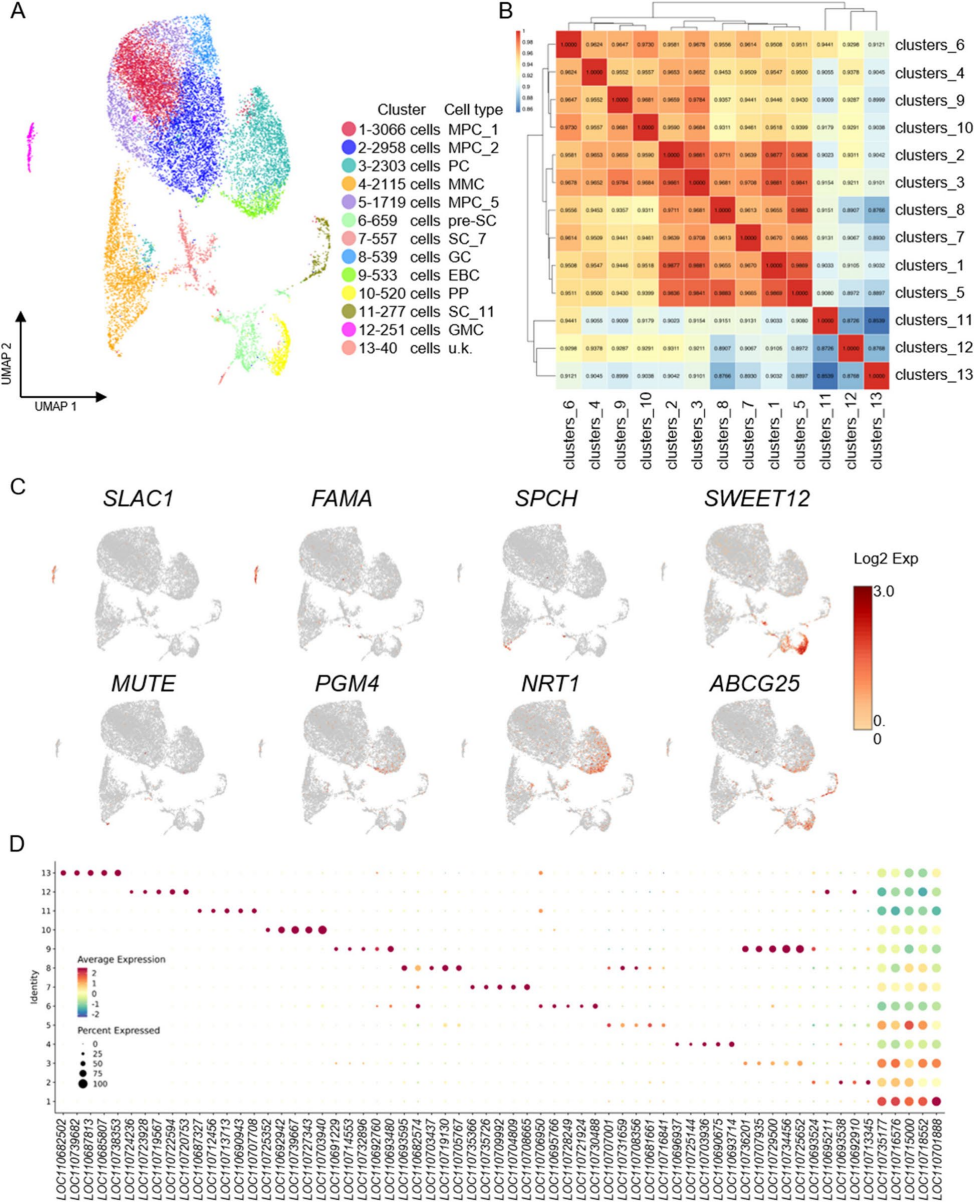
Identification of cell types and transcriptional profiles in two-week-old *quinoa* leaves using single-cell RNA sequencing. **A** UMAP plot displaying 13 identified cell types, each coded with different colors. Red arrows indicate the developmental trajectories of stalk cells. **B** Heatmap illustrating the correlation of gene expression between different cell clusters. The horizontal and vertical coordinates represent the different cell clusters. The numbers on the graph are Pearson correlation coefficients, with darker colors indicating higher degrees of correlation. **C** Visualization of known marker genes in the UMAP clustering maps. **D** Expression patterns of the top five differentially expressed genes (DEGs) with the highest expression levels in each sub-cluster. The average expression level (color) and the proportion of cells expressing each gene (size) are shown for each gene and cluster. Abbreviations: MPC, mesophyll cell; PC, pavement cell; MMC, meristemoid mother cell; pre-SC, pre-stalk cell; SC, stalk cell; GC, guard cell; EBC, epidermal bladder cell; PP, phloem parenchyma; GMC, guard mother cell; u.k., unknown

To investigate the development of the salt bladder, we performed single-cell RNA sequencing on the true leaves of two-week-old *quinoa* seedlings to identify and characterize the cell types and the expression patterns of genes that are especially expressed in each of them. Protoplasts were prepared by enzymatic digestion of leaves, filtered and screened through a 40 μm pore cell filter, and then used for sequencing library preparation. After quality assessment of the sequencing data, 15,985 high-quality cells were obtained, with a mean number of 8,766 UMI (unique molecular identifiers) per cell and 2,720 expressed genes per cell. The final number of cells was 15,537 after eliminating double-cells, multicell, and apoptotic cells, with a mean number of 8,138 UMI per cell and 2,626 expressed genes per cell (Table S1). Subsequently, we employed Uniform Manifold Approximation and Projection (UMAP) for dimensionality reduction and executed cluster classification analysis on the filtered single-cell sequencing data, which were classified into 13 cell clusters based on the similarity of specifically differentially expressed genes (DEGs) in each cluster (Fig. 3A and Table S2). The correlation between differentially expressed cell clusters was obtained by calculating the Pearson correlation coefficients of the mean values of gene expression between cell clusters. We found an extremely strong correlation between clusters 1, 2, and 5, which implies that they most likely belong to similar cell types (Fig. 3B). The expression distribution characteristics of representative marker genes in specific cell clusters were illustrated, highlighting top five DEGs expressed in specific cell cluster (Fig. 3C). To annotate the cell types and identify the new marker genes in *quinoa* leaves, we screened cluster-specific expressed genes in each cluster (Fig. 3D and Table S3). In plants, especially *quinoa*, known marker genes for identifying cell types remains limited and have been mainly identified in the model plant *Arabidopsis thaliana* so that homologous marker genes from other species can be used for cell type identification. In addition, since some known marker genes may be expressed in different cell types, although at different levels, we utilized the expression patterns of multiple marker genes to collectively identify different cell types in the true leaves of *quinoa*. The *Arabidopsis* homologs *basic helix-loop-helix* (*bHLH*) protein/*LOC110732543*, *MUTE*/*LOC110727789*, *TOO MANY MOUTHS* (*TMM*/*LOC110698887*). *SPEECHLESS* (*SPCH*/*LOC110725693*) are all specifically expressed in cluster 4 and are considered marker genes for meristemoid mother cells (MMC) (Fig. 3C) (Liu et al. 2020; Pillitteri et al. 2007). The *Arabidopsis* homolog *SUGARS WILL EVENTUALLY BE EXPORTED TRANSPORTERS 12* (*SWEET12*/*LOC110703940*) is a marker gene for phloem parenchyma (PP) and is highly expressed in cluster 10 (Fig. 3C) (Kim et al. 2021). The *Arabidopsis* homolog *FAMA*/*LOC110716316*, a marker gene for guard mother cell (GMC), is specifically highly expressed in cluster 12, and *AtFAMA* has a role in controlling the transition from GMC to guard cells (Fig. 3C) (Ohashi-Ito & Bergmann 2006). The *Arabidopsis* homolog *SLOW ANION CHANNEL-ASSOCIATED 1* (*SLAC1*/*LOC110702556*) is highly expressed in cluster 12, and *AtSLAC1* has been reported to encode a multi-transmembrane protein involved in the regulation of stomatal lineage cell development (Fig. 3C) (Deng et al. 2021). The *Arabidopsis* homolog *LIGHT HARVESTING CHLOROPHYLL A/B-BINDING* PROTEIN/*LOC110715346*, which is specifically expressed in clusters 1,2, and 5, has been reported to encode a chloroplast protein and to be involved in the regulation of stomatal development in the mesophyll cell (MPC) with high expression (Liu et al. 2020). Probable cation transporter *HKT6*/*LOC110738464* is specifically expressed in clusters 3, 9, and 11, and is associated with *HKT1*-type transporters (*high-affinity potassium transporter1*) belonging to the *HKT* family of transcription factors. *AtHKT1* plays a key role in the dynamic balance of Na⁺ and K⁺ under salt stress and contributes to the reduction of Na⁺-specific toxicity in plants (Ali et al. 2019). *NITRATE TRANSPORTER 1* (*NRT1*/*PTR FAMILY 3.1*-*like*/*LOC110719467*) is highly expressed in clusters 3 and 9 (Fig. 3C), and studies have reported that *NRT1* is involved in nitrate uptake, partitioning, and storage in higher plants (Ali et al. 2019). *ATP-binding cassette family G25* (*ABCG25*/*LOC110713974*) is specifically expressed in clusters 6, 7, 9, 10 and 11 (Fig. 3C). *AtABCG25* is a cytosolic ABA transporter protein, and at the same time, ABCG transporter proteins are involved in stratum corneum lipid transport (Kuromori et al. 2016; McFarlane et al. 2010). It has been shown that SCs contain a large number of liposomes and a thick stratum corneum enriched with genes related to ‘lipid metabolism’ (Bazihizina et al. 2022). S*hort chain-dehydrogenase/reductases* (*SDR*/*LOC110736399*) are specifically expressed in clusters 4, 6, and 9, and salt stress in *quinoa* can be alleviated by up-regulated expression of SDR genes (Al-Mushhin et al. 2021).

### Annotation of cell types based on the analysis of GO enrichment and KEGG

**Fig. 4:**
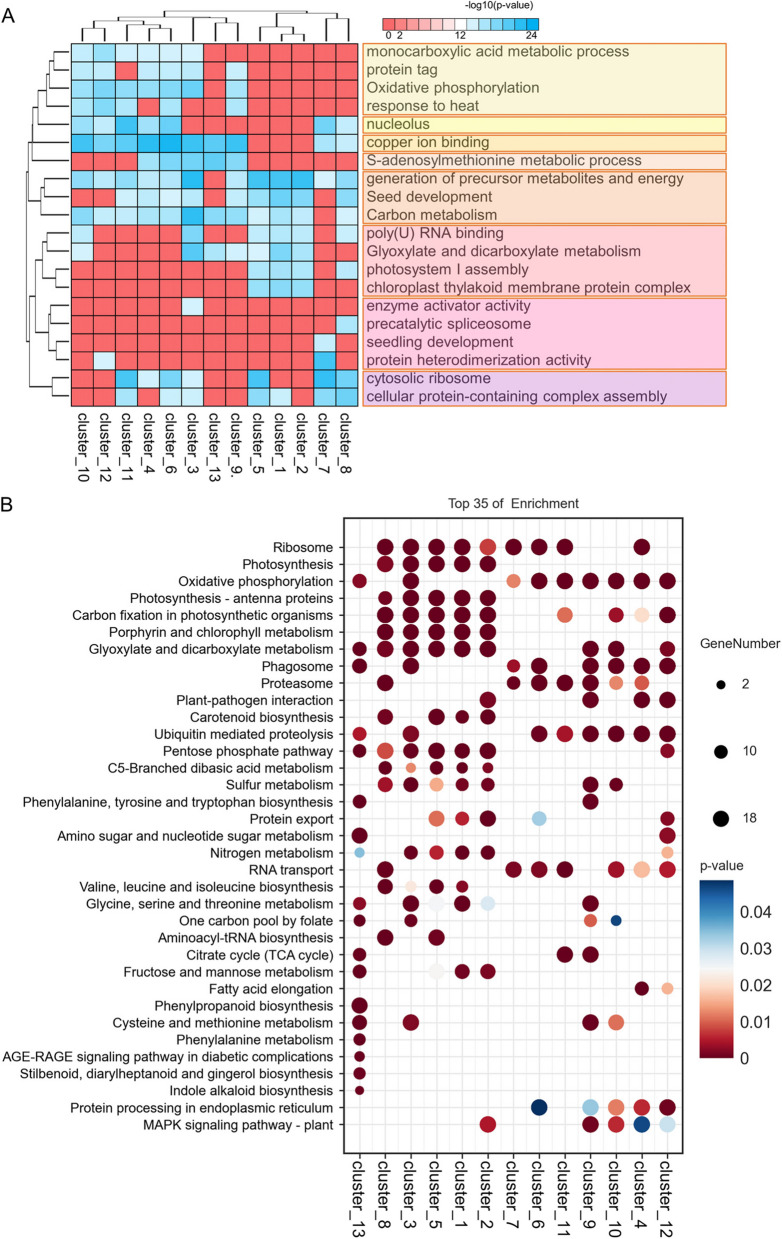
Enrichment analysis of differentially expressed genes (DEGs) in *quinoa* leaf cell clusters. **A** Gene Ontology (GO) enrichment analysis of DEGs across 13 cell clusters. **B** Kyoto Encyclopedia of Genes and Genomes (KEGG) pathway analysis of DEGs across 13 cell clusters

Due to the lack of studies on cell types in *quinoa* leaves at the single-cell level, there is no resource of known marker genes for identifying cell types. For those cell types that could not be identified with available marker genes, we tried to annotate the cell cluster with the information of the Gene Ontology (GO) and Kyoto Encyclopedia of Genes and Genomes (KEGG) pathway (Fig. 4 and Table S4-5). Interestingly, we found that the DEGs of clusters 1, 2, and 5 are enriched in very similar GO terms (Fig. 4A) and KEGG pathway (Fig. 4B), consistent with the Pearson correlation between cell populations from the above analysis (Fig. 3B). The GO terms enriched in clusters 1, 2, and 5 include mainly the generation of precursor metabolites and energy, photosystem I assembly, and chloroplast thylakoid membrane protein complex (Fig. 4A); the KEGG pathways enriched in these clusters are photosynthesis and porphyrin and chlorophyll metabolism (Fig. 4B). Similarly, cluster 8 is enriched for GO terms related to photosystem I assembly and KEGG pathways related to porphyrin and chlorophyll metabolism. In contrast, clusters 4 and 12 are not enriched for “photosynthesis-related”, which is consistent with the lack of chloroplasts and photosynthetic functions in MMC and GMC. The DEGs of cluster 9 are enriched for endocytosis, a typical feature of EBC (Fig. S4A). In the KEGG pathway analysis, it was observed that clusters 9 and 10 are enriched in similar pathways. The gigantic vesicle in mature salt bladders caused chloroplast organelles to be pushed to the edge of the cell, resulting in a deprivation of photosynthetically relevant capabilities. Based on this observation, it is hypothesized that cluster 10 consists of phloem parenchyma (PP) cells, which are responsible for the carbon fixation function in photosynthetic tissues (Fig. 4B). Cluster 9 is enriched to the phagosome, proteasome, and ubiquitin-mediated proteolysis (Fig. 4B). Clusters 6, 7, and 11 are enriched in the similar KEGG pathway (Fig. 4B), and their GO terms are enriched in cytosolic ribosomes, suggesting that these cell clusters are associated with protein translation in the cytoplasm. This is consistent with our electron microscopy observation of dense cytoplasm, enriched cytosolic ribosome, and vigorous protein translation activity in SC. The KEGG pathway analysis of SC cluster 6 reveals enrichment for protein processing in the endoplasmic reticulum and protein export (Fig. 4B). These functions are typically associated with cells in the early stages of development. In contrast, clusters 7 and 11 do not show enrichment in these pathways. Therefore, it is hypothesized that cluster 6 represents pre-SC (pre-stalk cell), while clusters 7 and 11 correspond to SC_7 and SC_11. To distinguish between these two different stalk cells, they are labeled with the cell cluster they belong to. In summary, based on the marker genes screened in each cell cluster, as well as the results of GO enrichment analysis and KEGG pathway analysis of these cell clusters, we deduced that clusters 1, 2 and 5 belong to MPC_1, MPC_2, MPC_5, cluster 4 belongs to MMC, cluster 8 belongs to GC, cluster 9 belongs to EBC (epidermal bladder cell), cluster 10 belongs to PP, and cluster 12 belongs to GMC.

In the KEGG pathway analysis, we found that DEGs associated with seleno-compound metabolism, Phagosome, ubiquitin-mediated proteolysis, and Sulfur metabolism are enriched in EBC (cluster 9) (Fig. 4B). For example, *phosphoglycerate mutase-like* *protein 4*/ *LOC110682269* was specifically expressed in EBC (cluster 9) (Fig. S4B), and it was reported that its *Arabidopsis* homolog *AT3G50520* encodes a phosphoglycerate mutase-like family of proteins, which are involved in the metabolic process in response to inorganic substances and sulfur (Jedrzejas 2000). Similarly, cluster 3 is also enriched for Selenocompound metabolism and Nitrogen metabolism. In GO analysis, we found that EBC (cluster 9) and cluster 3 are enriched for similar functions, including oxidative phosphorylation, copper ion binding, s-adenosylmethionine metabolic process, and carbon metabolism, phosphorylation, copper ion binding, S-adenosylmethionine metabolic process, and Carbon metabolism (Fig. 4A). Surprisingly, cluster 3 was also enriched for the cytosolic ribosome-associated with cytoplasmic solute transport. This suggests that cluster 3 may be a pavement cell (PC) adjacent to salt bladders. In the GO analysis of DEGs (Fig. 4A), we found that DEGs in pre-SC (cluster 6) and PP (cluster 10) were enriched in similar GO terms, copper ion binding, response to temperature stimulus, carbon metabolism, oxidative phosphorylation, and significant enrichment in the nucleolus. Therefore, we hypothesized that the pre-stalk is closely related to PP cells. Cluster 13 was named unknown (u.k.) because there were no marker genes that could identify the cell type in cluster 13 and the results of GO enrichment analysis and KEGG pathway analysis differed greatly from those of other clusters, making it impossible to determine its cell type. We chose the marker genes of MMC, pre-SC, SC, and EBC for feature map presentation since this is the first instance of single-cell annotation of *quinoa* salt bladder cell types (Fig. S5).

### Analyzing the developmental trajectory of the EBC in the leaves of *quinoa* by pseudo-time series analysis

**Fig. 5:**
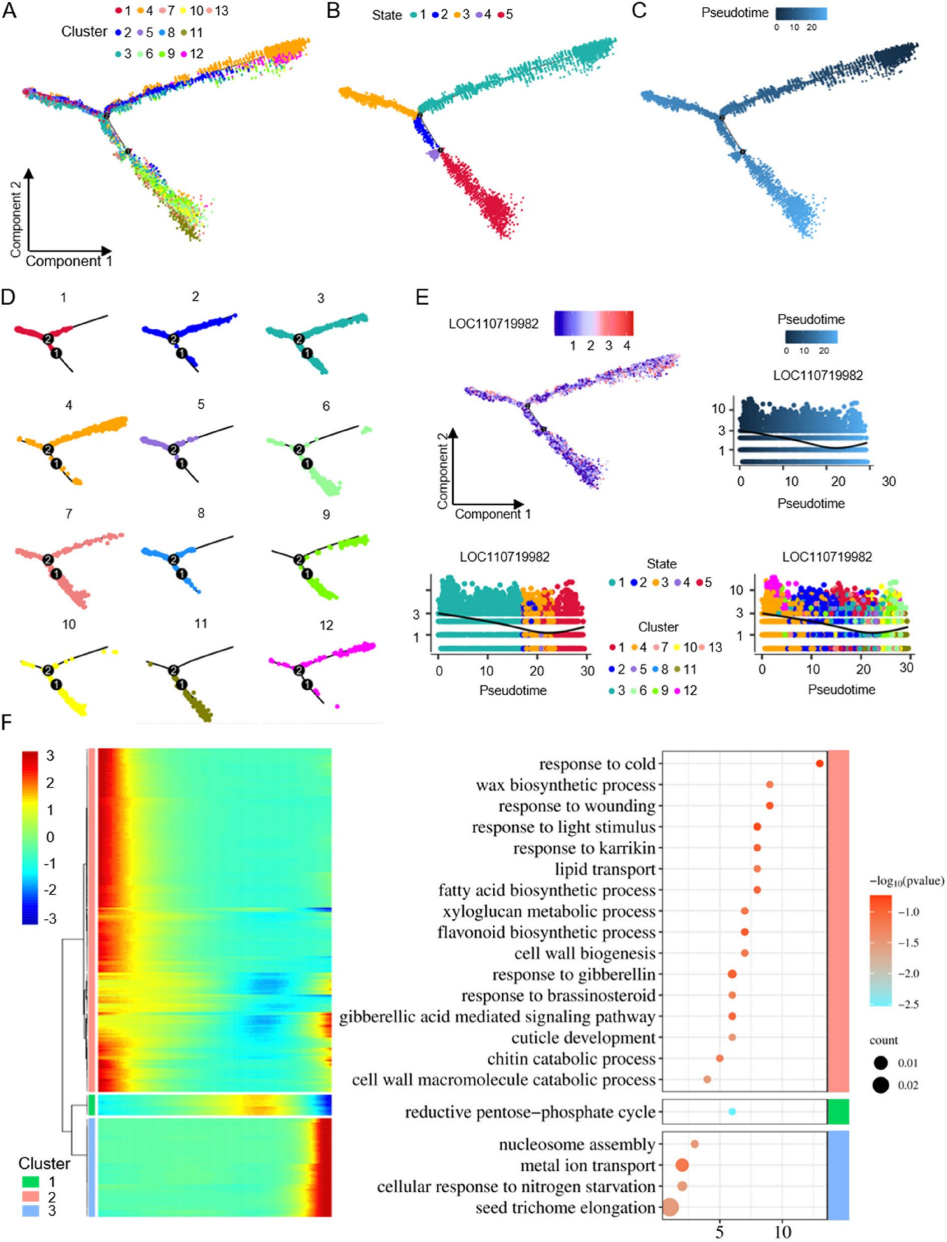
Pseudotemporal analysis of *quinoa* leaf cell clusters. **A**-**C** Monocle2 was used to simulate the developmental trajectories and differentiation states of *quinoa* leaf cell clusters over pseudotime, with each dot representing a single cell. **D** The distribution of various cell types along these pseudotime trajectories is shown, with each dot color-coded by cell type. **E** The expression profiles of the most variable gene along pseudotime were identified using Monocle2’s differential Gene Test function (q-value < 0.01). The black line represents the overall expression trend, with each dot indicating a single cell. **F** A heatmap displays the genes with highly variable expression across three gene modules along pseudotime. Additionally, a dot plot presents the GO enrichment analysis of these highly variable genes

To analyze the temporal distribution of the evaluated single cells, a pseudo-time trajectory was employed to visualize how cells from each cluster were distributed along the main stem using the Monocle 2 (Trapnell et al. 2014). The proposed temporal path of the *quinoa* leaf samples had three branches distributed along a major developmental trajectory, with different clusters of cells aligned more clearly at different branch positions in the pseudo-temporal path (Fig. 5A, C). Pseudotime analysis revealed that all single cells were divided into five states of leaf cell development and differentiation state (Fig. 5B-C). Constructing a pseudo-temporal trace of each cell cluster based on cell cluster coloring can help in visualizing the developmental timing of each cluster (Fig. 5D). Overall, the developmental progression of *quinoa* salt bladder cells was from MMCs to EBCs and SCs. Interestingly, we found that cell cluster 7 appeared earlier than cell cluster 11 on the pseudotemporal curve, so the stalk cell cluster developmental order was pre-SC_6 to SC_7 to SC_11 (Fig. 5D). Illustration of the pseudotime expression pattern of the top 1 gene with highly variable expression showed *LOC110719982* exhibits high expression levels at the onset of differentiation and remains highly expressed in MMC (Fig. 5E). In principle, the distribution characteristics of different types of cells on the developmental trajectory can initially determine the relationship between these cells at different developmental stages. The distribution of GCs in the developmental trajectory is relatively concentrated, but MPC_2 and PCs can be found at several time points on the developmental trajectory, suggesting that the development of MPCs and PCs is more complex.

The pseudo-time heatmaps of the top 300 genes with highly variable expression show that their pseudo-time patterns can be divided into three modules (Fig. 5F). In module 1, the expression of most genes first increases and then decreases along the trajectory of pseudo-time (Fig. 5F). GO enrichment of module one reveals genes involved in cell wall biogenesis, and response to gibberllin and brassinosteroid (Fig. 5F and Table S6). In module 2, the expression of all genes gradually decreases, except for a small number of genes that reach high levels of expression at the end of the trajectory of pseudo-time (Fig. 5F). In module 3, the expression of all genes remains at low and stable levels but increases to its highest level in the final stage of the trajectory (Fig. 5F). GO enrichment of module three revealed genes involved in metal ion transport, and seed trichome elongation (Fig. 5F and Table S6).

**Fig. 6:**
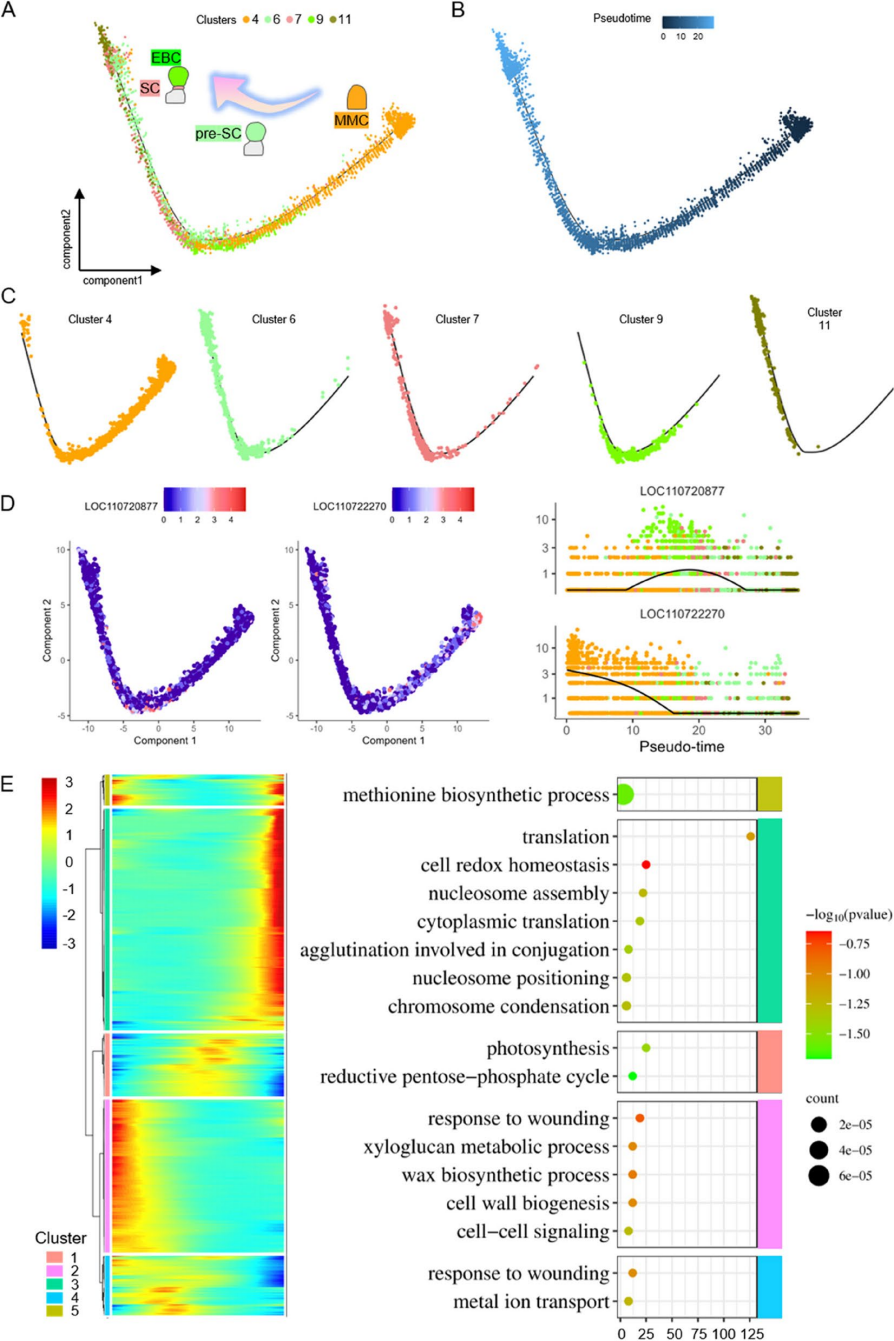
The development trajectory of EBC from MMC. **A**-**B** Monocle 2 was used to simulate the developmental trajectory of EBC over pseudotime, with each dot representing a single cell. **C** The distribution of different EBC-associated cell types along these pseudotime trajectories is shown, with each dot color-coded by cell type. **D** The expression profiles of the two most variable genes along pseudotime were identified using Monocle 2’s differential Gene Test function (q-value < 0.01). The black line represents the overall expression trend, with each dot indicating a single cell. **E** A heatmap displays genes with highly variable expression across five gene modules along pseudotime. Additionally, a dot plot presents the GO enrichment analysis results for these highly variable genes

To decode the developmental trajectory from MMC to EBC, the cells of MMC, pre-SC, SC, and EBC were extracted and re-analyzed in a quasi-chronological manner. Cell developmental trajectories indicate that MMC is situated at the starting point of differentiation, and then differentiates into pre-SC, which further differentiates into SC and EBC (Fig. 6A-C). The quasi-chronological expression profile of the top 2 genes with hypervariable expression showed that *LOC110720877* is highly expressed in EBC, and *LOC110722270* in MMC in the early stages of differentiation (Fig. 6D). The top 2,000 hypervariable genes were selected for quasi-chronological cluster analysis, which was divided into 5 modules (Fig. 6E). Module 2 is expressed at the early stage, and the GO enrichment results indicate the xyloglucan metabolic process, wax biosynthetic process, cell wall biogenesis, and cell–cell signaling (Fig. 6E and Table S7). Modules 1 and 4 include genes expressed at the mid-term, and the GO enrichment results indicate photosynthesis, reductive pentose-phosphate cycle, and metal iron transport (Fig. 6E and Table S7). Genes of module 3 are expressed at the later stage, and the GO enrichment results reveal cell redox homeostasis, nucleosome assembly, agglutination involved in conjugation, nucleosome positioning, and chromosome condensation (Fig. 6E and Table S7). Module 5 is expressed at both early and late stages, and the GO enrichment results indicate the methionine biosynthetic process (Fig. 6E and Table S7).

### Salt stress promotes the development of salt bladders in *quinoa*

GO enrichment analyses showed significant enrichment of terms responsive to salt stress in SC and EBC clusters. In addition, we identified several marker genes in stalk and salt bladder cell clusters in response to salt stress, such as *bHLH112-like* (*LOC110693480*), *LOC110707708*, *LOC11069094*, and *LOC110705347*. Among them, it was reported that *AtbHLH112* belongs to subfamily F and responds to abiotic adversity through increased proline levels and enhances ROS scavenging thus enhancing tolerance to abiotic stress (Liu et al. 2015). The observation that these genes related to salt stress are highly expressed in SC and EBC cells suggests that salt stress has a potential role in influencing the development and function of salt bladders.

**Fig. 7:**
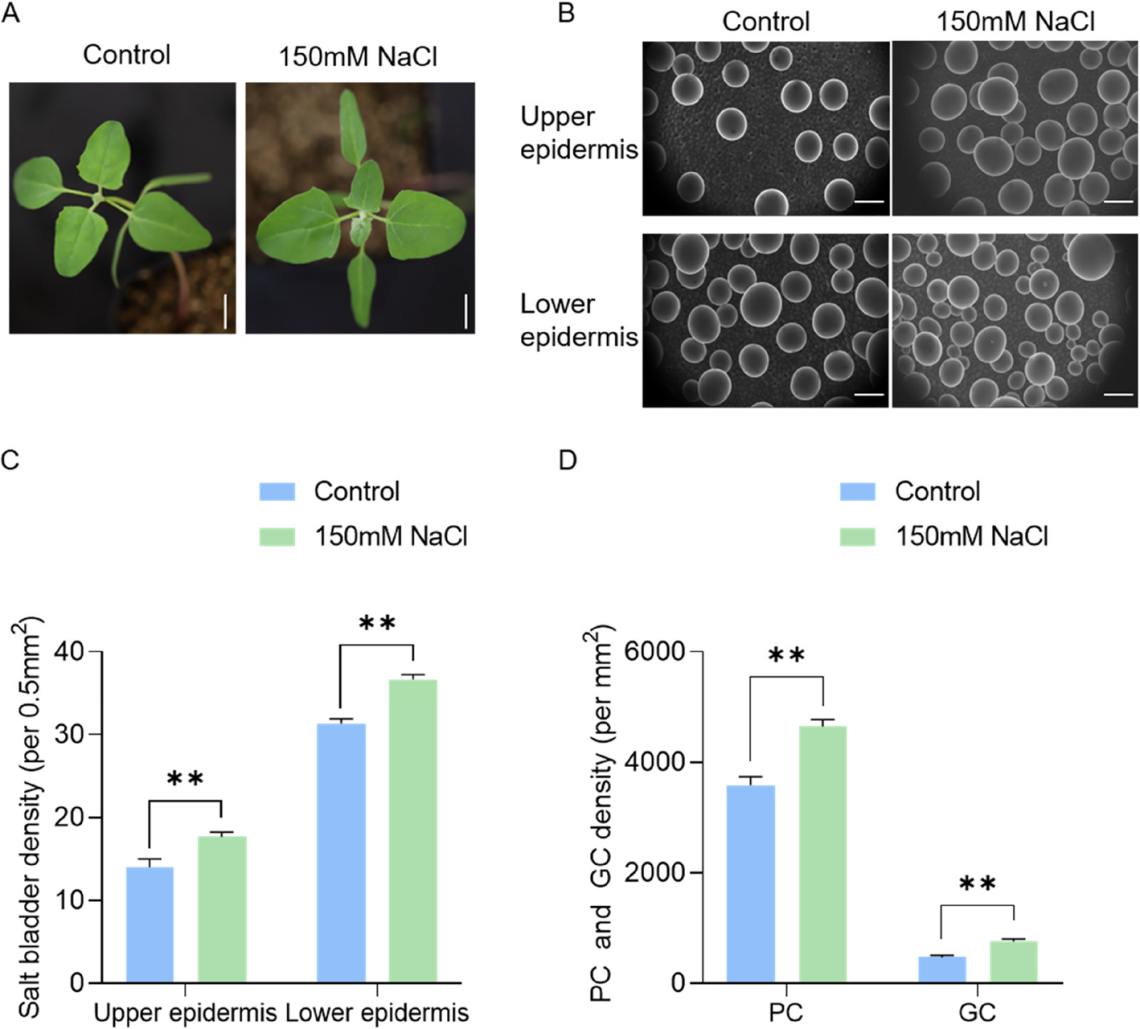
Effect of salt stress on the development of salt bladders and PCs in *quinoa* leaves. **A** The growth phenotype of *quinoa* under control conditions and after 2 weeks of 150 mM NaCl treatment; Scale bar: 1 cm. **B** SEM images illustrating the impact of salt stress on the density of salt bladders on the upper and lower epidermis of *quinoa* leaves; Scale bar: 100 μm. **C**-**D** The density of salt bladder, PC, and GC on the surface of *quinoa* under control and salt stress conditions (** *p* < 0.01, t-test)

To characterize the effect of salt treatment on the development of PC, GC, and EBC, we treated one-week-old *quinoa* seedlings with 150 mM NaCl for two weeks (Fig. 7A). It was observed that the growth rate of *quinoa* seedlings treated with salt was slightly lower compared to the control (Figs. 7A and 9A). Image J (Version 1.2.4, RRID:SCR_003070) software was employed to count the number and size of cells. Statistical analysis showed that the EBC density on the leaf surface increases significantly with salt treatment, especially in the lower epidermis (Fig. 7B, C). The density of PC and GC in the upper epidermis significantly increased after salt treatment (Fig. 7D). These results imply that Na^+^ stimulates the formation of salt bladders.

### Nitrogen promotes the development of salt bladders

KEGG pathway enrichment analysis revealed that nitrogen metabolism is significantly enriched in PC cell clusters, and sulfur metabolism is significantly enriched in both PCs and EBC (Fig. 4B). These results suggest that nitrogen metabolism and sulfur metabolism may have potential roles in influencing the development of salt bladders.

**Fig. 8:**
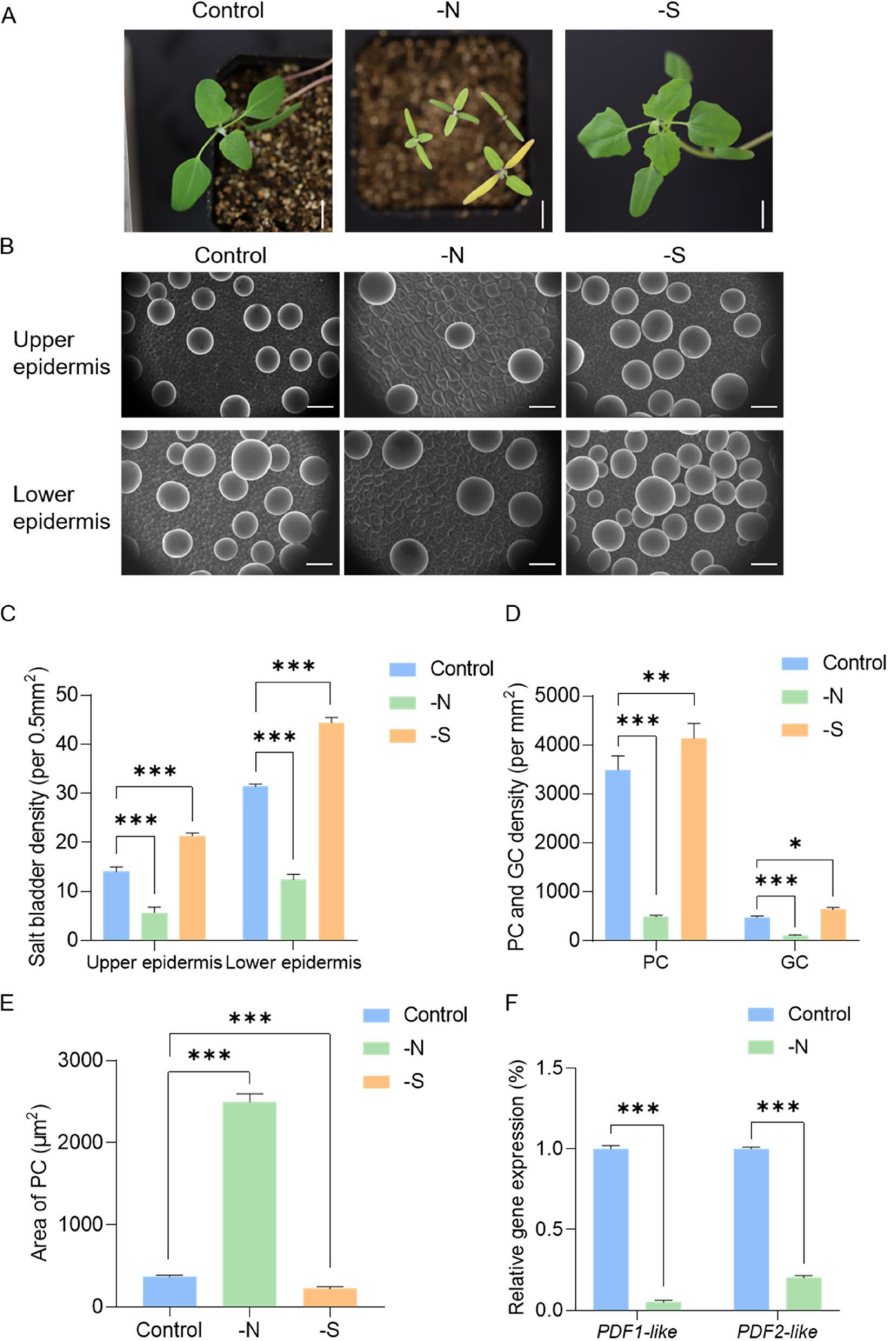
Effects of nitrogen and sulfur deficiency on the development of salt bladders and PCs in *quinoa* leaves. **A** The growth phenotypes of *quinoa* after two weeks of nitrogen and sulfur deficiency; Scale bar: 1 cm. **B** SEM images illustrating the impact of nitrogen and sulfur deficiency on the density of salt bladders on the upper and lower epidermis of *quinoa* leaves; Scale bar: 100 μm. **C**-**E** Changes in density of salt bladder cells, PC, GC, and size of PC under nitrogen deficiency and sulfur deficiency treatment (* *p* < 0.05, ** *p* < 0.01, *** *p* < 0.001, t-test). **F** The expression level of PDF1-like and PDF2-like decreases under nitrogen deficiency (*** *p* < 0.001, t-test)

To analyze the effects of nitrogen, and sulfur on the development of PC, GC, and EBC, we grew *quinoa* under nitrogen/sulfur deficiency for two weeks. Growth of *quinoa* was significantly inhibited, especially in the case of nitrogen deficiency (Fig. 8A). The number of salt bladders on the surface of *quinoa* increased in the absence of sulfur and decreased in the absence of nitrogen (Fig. 8A, B, C). This indicates that sulfur plays an inhibitory role in salt bladder formation while nitrogen positively regulates salt bladder formation. Statistical analysis of the number of PCs and GCs in the upper epidermis showed that nitrogen deficiency significantly decreases the number of PCs and GCs (Fig. 8B, D).

*ATML1* and *PDF2* are known to regulate epidermal cell development (Abe et al. 2003). Therefore, we examined the expression of the homologs of *ATML1* and PDF2 corresponding to *PDF2*-*LIKE* and *PDF1-LIKE*, respectively, under nitrogen deprivation. The results showed that both the *ATML1* and *PDF2* homologs which regulate the development of *quinoa* leaves, are repressed under nitrogen deficiency conditions (Fig. 8F). This repression leads to a disruption in the normal growth pattern, culminating in the abnormal enlargement of provascular cells (PCs) (Fig. 8E). Under conditions of sulfur deficiency, a pronounced increase in the density of PCs within the *quinoa* upper epidermis was observed (Fig. 8D). Additionally, there was a significant reduction in the individual area of PCs (Fig. 8E). In stark contrast, the sulfur-deficient treatment induces a significant increase in the GC count (Fig. 8D). These observations suggest a potential facilitative effect of nitrogen on and inhibitory role of sulfur in the developmental process of *quinoa* salt bladders.

### Salt stress-induced salt bladder development is independent of nitrogen signaling

**Fig. 9:**
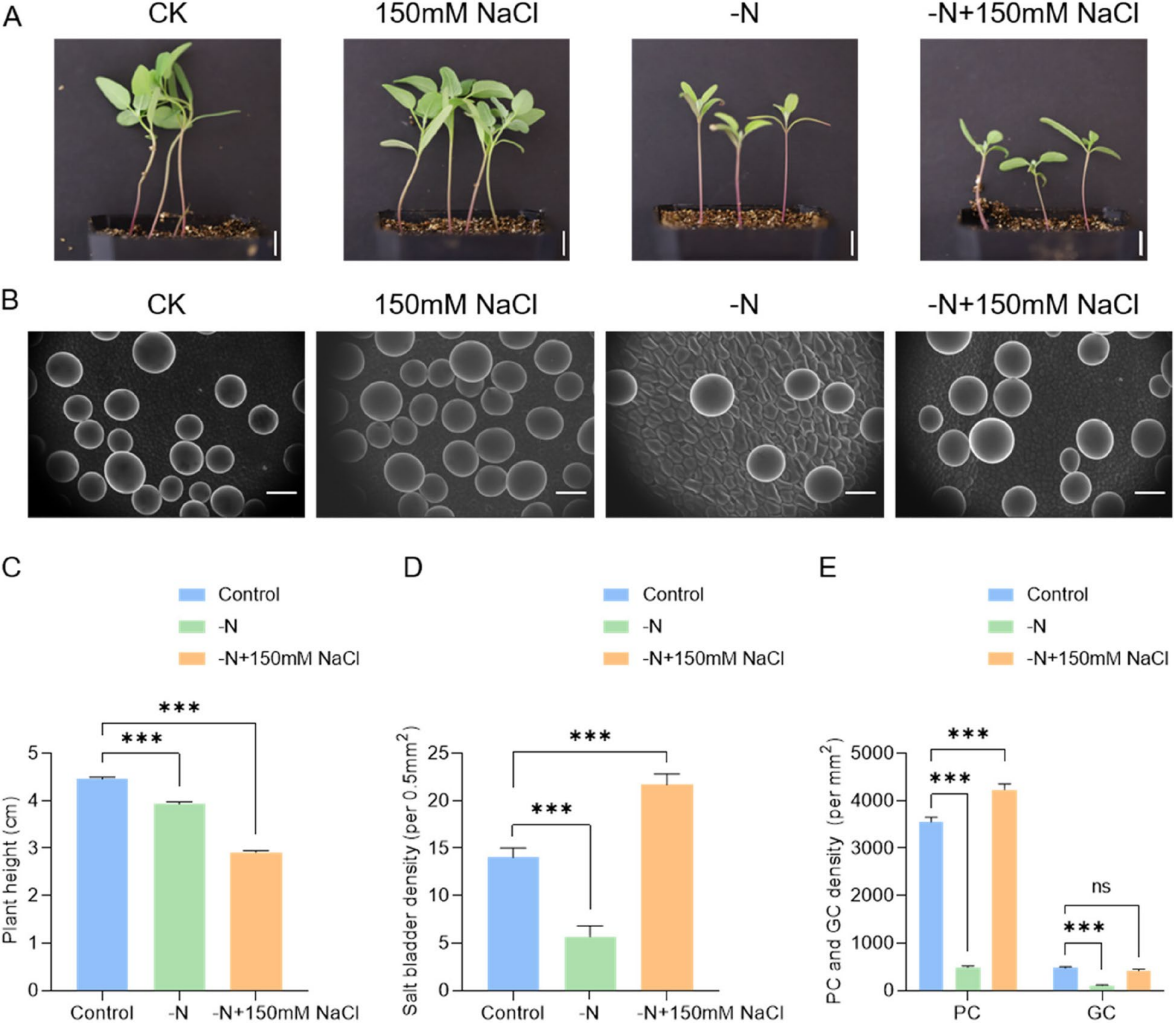
Effect of nitrogen deficiency and salt stress co-treatment on the development of salt bladders and PCs in *quinoa* leaves. **A** The growth phenotypes of *quinoa* after two weeks under 150 mM NaCl or nitrogen deficiency treatments; Scale bar: 1 cm. **B** SEM images illustrating the impact of 150 mM NaCl or nitrogen deficiency on the density of salt bladders on the upper epidermis of *quinoa* leaves; Scale bar: 100 μm. **C** Effects of nitrogen deficiency and salt stress co-treatment on *quinoa* plant height (*** *p* < 0.001, t-test); **D**-**E** Changes of density of salt bladder, PCs, and GCs after nitrogen deficiency with salt stress co-treatment

By observing salt bladders after salt stress, we found that high salt concentrations promote the development of salt bladders in *quinoa* leaves (Fig. 7B). To test whether high concentrations of Na^+^ could act as a signal to promote salt bladder development, we grew *quinoa* seedlings under salt stress and nitrogen deficiency. The results showed that the development and height of plants subjected to this combined treatment were significantly reduced compared with the control and plants subjected only to salt stress (Fig. 9A, C). SEM observation of leaves showed that the number of salt bladders was significantly increased by combined nitrogen deficiency and salt stress compared with nitrogen deprivation (Fig. 9A, B, D), suggesting that salt stress promotes the development of salt bladders under nitrogen deficiency and that the development of salt bladders induced by high concentrations of Na^+^ is independent of the nitrogen signals. In addition, statistical analyses of PCs revealed that the area of PCs in *quinoa* leaves co-treated with nitrogen deficiency and salt stress slightly decreased compared with the nitrogen deficiency treatment, partially restoring the phenotype of abnormally enlarged PCs induced by nitrogen deficiency (Fig. 9E). These results suggest that salt stress slightly restores the inhibitory effect on phototrophic growth caused by nitrogen deficiency. This may be a mechanism of adaptation to adverse growth conditions such as salinity and nitrogen deficiency which occurred during the evolution of *quinoa*.

### Construction of a regulatory network of transcription factors of pigment gland morphogenesis

**Fig. 10:**
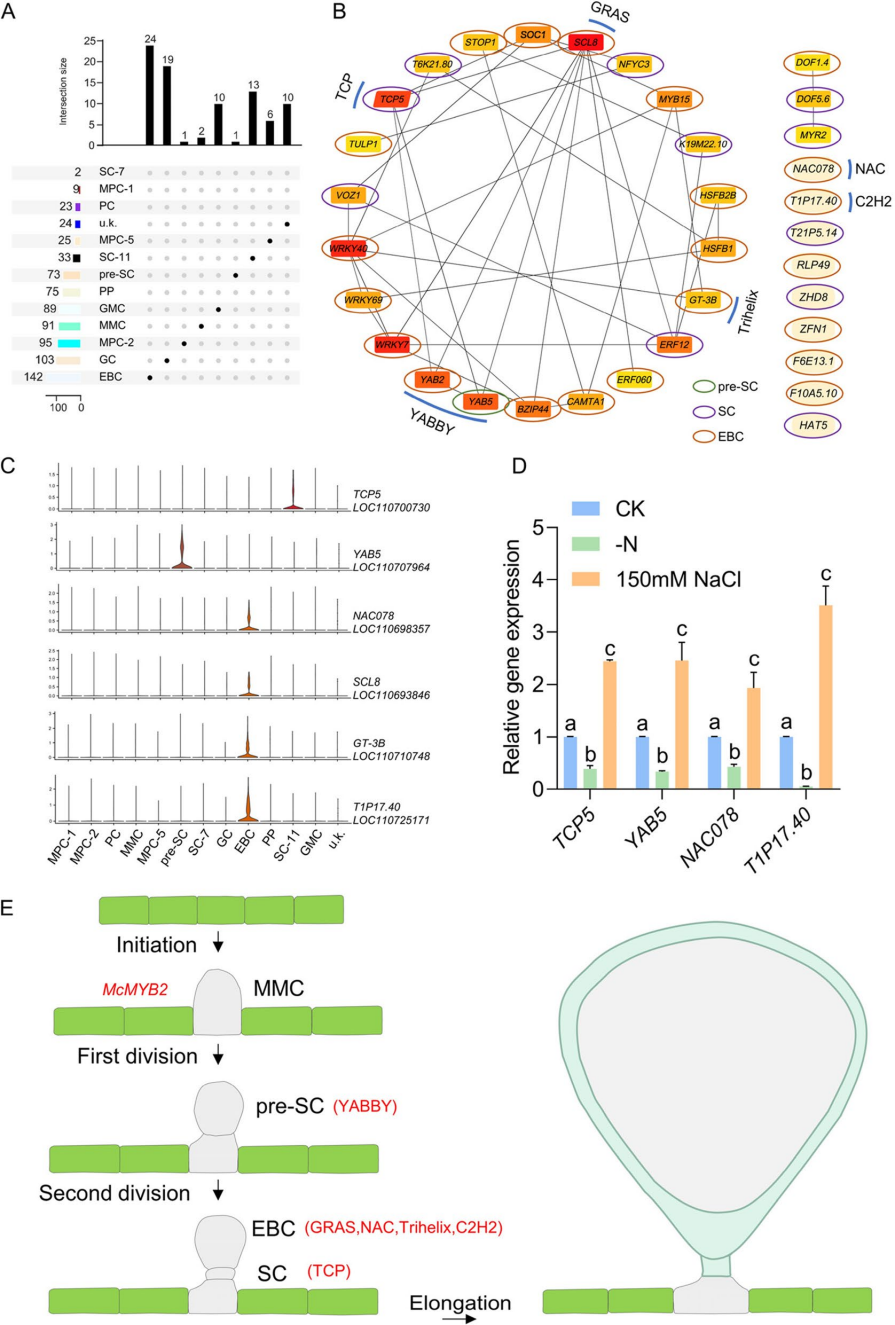
Transcription factors regulatory network of EBC development in *quinoa. ***A** Venn diagram that systematically categorizes the transcription factors enriched in each cell cluster. **B** Transcription factors regulatory network of EBC development inferred from pre-SC, SC, and EBC. **C** The violin plot provides a visual representation of the expression patterns for candidate transcription factors. **D** Gene expression of candidate transcription factors from pre-SC, SC, and EBCs after nitrogen deficiency and salt stress. **E** Proposed model of transcription factors for the regulation of EBC development

To elucidate the regulatory mechanisms of EBC development, we conducted a comprehensive analysis of the transcription factors of the different cell clusters. We searched for transcription factors among all differentially expressed genes and generated a diagram that systematically classified the transcription factors enriched in each cell cluster. Two transcription factors were retrieved in SC-7, but not exclusively expressed in SC-7 (Fig. 10A and Table S8). There are 33 transcription factors were retrieved in SC-11, of which 13 were specifically expressed in SC-7 (Fig. 10A and Table S8). There are 73 transcription factors were retrieved in pre-SC, of which one was specifically expressed only in pre-SC (Fig. 10A and Table S8). There are 142 transcription factors were retrieved in the EBC, of which 24 were specifically expressed in the EBC (Fig. 10A and Table S8). We used STRING (https://cn.string-db.org/) to predict the interactions between the above transcription factors and visualize the interaction network via cystoscope (Fig. 10B and Table S9). Based on functional annotations of transcription factors specifically expressed in pre-SC, SC, and EBC, 6 candidate transcription factors were screened for possible involvement in salt bladder development (Fig. 10C). *TCP5* directly promotes the transcription of *KNAT3* and indirectly activates the expression of *SAW1*. Earlier studies also showed that *TCP5* regulates *KNAT3* and *SAW1* in a temporal- and spatial-specific manner during the formation of serrations (Yu et al. 2021). The *YABBY* gene family, predominantly expressed in lateral organs with polarity, is essential for leaves to initiate as dorsiventral structures and subsequently activate the developmental pathways crucial for lamina formation (Sarojam et al. 2010). In particular, the failure to establish a marginal leaf domain prevents the initiation of *CINCINATTA class TCP* genes (*CIN-TCPs*) and leads to the reactivation of shoot apical meristem (SAM) specific developmental programs (Sarojam et al. 2010). Transcription of *PI4Kγ5* is relatively high at the early stage and decreases along with the leaf development, and is finally restricted at the leaf margin, especially at the serration tips of mature leaves, which is consistent with *PI4Kγ5* regulating cell division at the leaf margin by regulating auxin synthesis. It is hypothesized that *PI4Kγ5* interacts with membrane-bound *ANAC078* to promote its proteolytic processing, possibly through phosphorylation, to maintain the normal auxin concentration, and hence regulate the final leaf shape with weak serrations. Deficiency of *PI4Kγ5* results in the defective interaction and loss of *ANAC078* cleavage, resulting in enhanced auxin synthesis and promoting cell proliferation at leaf teeth with highly deep serrations (Tang et al. 2016). The repression of *BRON* (*BRONTOSAURUS*), encoding a *C2H2*-like zinc finger transcription factor, lifts its repression on the cyclins *CYCD3;1* and *CYCP4;*1, leading to BR-induced cell division (Clark et al. 2021). The foliar application of GA_3_ increased the expression of *GT-3b* and salt tolerance (Wang et al. 2020), trichome developmental selectors *GLABRA3* (*GL3*) and *GLABRA1* (*GL1*), *encoding basic helix-loop-helix* (*bHLH*) and *MYB* transcription factors. Several of the *GL3/GL1* direct targets are expressed early during trichome formation, including the transcription factors *MYC1* (*bHLH*), and *SCL8* (*GRAS*), associated with trichome formation (Morohashi & Grotewold 2009). Since the application of NaCl promotes the development of EBCs and nitrogen deficiency inhibits their development, we examined the expression of candidate transcription factors under different treatments. The expression of transcription factors *TCP5*, *YAB5*, *NAC078*, and *T1P17.40* was significantly increased with 150 mM NaCl, and conversely, their expression was significantly decreased under nitrogen deficiency (Fig. 10D). *YABBY* family transcription factors promote the formation of pre-SC while *GRAS*, *NAC*, *Trihelix*, and *C2H2* family transcription factors promote the formation of *EBC*. *TCP* family transcription factors promote the formation of SC (Fig. 10E). These results suggest that transcription factors *TCP5*, *YAB5*, *NAC078*, *SCL8, GT-3B,* and *T1P17.40* may play an important role in the regulation of EBC development.

## Discussion

### Single-cell analysis of salt bladder development in *quinoa*

The differentiation of salt bladders is irreversible and accompanied by dramatic changes in organelles and cellular morphology. Using SEM and TEM, we observed dynamic changes in the shape of intracellular subcellular organelles during salt bladder development. Using single-cell transcriptomic techniques, we could study the interplay of *quinoa* salt bladder-specific developmental regulatory programs and the impact of external environmental factors on the fate decisions of different cell types from a single-cell perspective. The combination of marker genes identified in past studies and GO analysis enable us to reliably classify and define cell types. Furthermore, a series of novel marker genes were identified across various cell types. Additionally, we investigated the impact of minerals and NaCl on the development of salt bladder cells by examining their development patterns in *quinoa* leaves under conditions of nutrient deficiency and salt treatment.

By SEM of true leaves of *quinoa* seedlings (Fig. 1), we observed that the development of salt bladders undergoes a special differentiation process, which leads to the gradual expansion of their apical vesicles and the accumulation of salt. We used transmission electron microscopy to observe salt bladders at different stages of development (Fig. 2) and found that during salt bladder development, the primary epidermal cells undergo pendulous pericycle divisions to form SCs and EBCs. The EBCs, through plasmodesmata, continuously absorb vesicles and transport vesicles from the periphery of the epidermal cells, so that their vesicles increase in number continuously, and eventually almost fill up the whole salt bladder cells. As a result of this process, organelles such as Golgi and mitochondria are squeezed to the edge of the salt bladder cells. The nucleus in the stalk cells, together with the surrounding endoplasmic reticulum and Golgi, promotes the transport of substances in the epidermal cells and salt bladders. The epidermal cells are dense, with a large number of chloroplasts, mitochondria, endoplasmic reticulum, and vesicles, which provide the energy and carriers for the transport of substances.

The development of salt bladder cells in *quinoa* is influenced by various critical factors including salinity, temperature, and metabolites (Xie et al. 2022). Previous studies, mostly focused on the whole plant, could not clearly distinguish the specific functions of these factors in different cell types. For example, low salinity promotes *quinoa* growth whereas high salinity inhibits *quinoa* growth, suggesting that *quinoa* has a very efficient osmoregulatory system to adapt to sudden increases in salt stress (Hariadi et al. 2011). Utilizing DEGs from distinct cell clusters combined with GO analysis, we identified genes potentially involved in regulating the development of salt bladder cells. For example, GO heatmap analysis showed that genes preferentially expressed in SC and EBC were mainly involved in S-adenosylmethionine metabolic processes, copper ion binding, and oxidative phosphorylation (Fig. 4A). Notably, genes highly expressed in SC were involved in the formation of cytoplasmic ribosomes. Stalk cells lack vesicles, but are dense with an abundant endoplasmic reticulum and Golgi apparatus to provide carriers for material transport (Otterbach et al. 2021). This suggests that genes expressed in SCs may be involved in protein synthesis during solute transport. GO heatmap analysis also showed that MPC_1, MPC_2, and MPC_5 participated in relatively similar GO processes (Fig. 4A). Primarily, these processes involve precursor metabolite synthesis and energy production, which implies an intricate interplay among these cell types regarding gene expression and their respective cellular functions. The analysis of transcription factor interactions within specific cell types is crucial for understanding the regulatory mechanisms that govern cellular differentiation and function. Analysis of interactions of transcription factors specifically expressed in pre-SC, SC, and EBCs suggests that *TCP5*, *YAB5*, *NAC078*, *SCL8*, *GT-3B,* and T1P17.40 may play important roles in the developmental regulation of EBCs (Fig. 10E). *TCP5*, a member of the *TCP* family of transcription factors, is known for its role in regulating gene expression related to cell cycle progression and organ development (Yu et al. 2021). During petal development, the process of cell division operates as a built-in timing mechanism that propels the transition from cell division to cell enlargement. And transcription factor *TCP5* is central to regulating this transition (Huang & Irish 2015). *TCP5*, a key regulatory factor in plant developmental processes, potentially plays a crucial role in modulating the development of stalk cells in* quinoa’s* salt bladders (Huang & Irish 2024). Interestingly, within the context of stalk cells, the presence of *TCP5* potentially curbs additional cell division and expansion. This implies that *TCP5*’s expression could act as a developmental switch, transitioning cells from a phase of growth to one of maturation. The precise regulation of *TCP5* is thus likely pivotal for the proper development of both petals and stalk cells, highlighting the complexity of gene expression patterns in orchestrating plant organogenesis. The *YABBY* gene family plays a crucial role in plant development, particularly in the specification of abaxial cell fate. YABBY genes play a pivotal role in the regulation of plant development by repressing the expression of *knotted1-like* *homeobox* (*KNOX*) homeobox genes, which in turn limits meristem formation and maintains distinct growth zones within the plant’s structural organization (Kumaran et al. 2002). A recent study reveals the molecular mechanism by which KNOX1 regulates node-internode morphogenesis in stems through antagonistic and synergistic interactions with the YABBY gene (Tsuda et al. 2024). *YAB5*, a member of the *YABBY* family, is implicated in the regulation of cell polarity and differentiation which are critical for the establishment of cell identity and tissue patterning (Sarojam et al. 2010). The overexpression of *YABBY1* in both tobacco and *Arabidopsis* plants led to a marked increase in trichome density on the epidermis, indicating that *AaYABBY1* may play a crucial role in trichome development and regulation of *Artemisia argyi* (Cui et al. 2024). *NAC078*, a member of the *NAC* family of transcription factors, is associated with various developmental processes, including the regulation of cell differentiation and the response to environmental stimuli (Tang et al. 2016). *GT-3B* is involved in the modulation of gene expression related to cell growth and differentiation (Wang et al. 2020). *SCL8* is essential for the maintenance of stalk cell identity and the regulation of asymmetric cell division (Morohashi & Grotewold 2009). Our findings underscore the significance of these transcription factors in the developmental trajectory of EBCs. Further research is warranted to elucidate the precise molecular mechanisms by which these factors interact and to determine their individual and collective contributions to the regulation of cell fate decisions. This knowledge will be instrumental in advancing our understanding of plant development and may have implications for the manipulation of plant growth and regeneration processes for agricultural and biotechnological applications.

### Salt stress promotes the development of salt bladder and PC in *quinoa*

After the salt bladder matures or is stimulated by external forces such as wind or rain, the bladder -like cells rupture, excreting salt out of the body, thus reducing the damage of salt to plants (Shabala et al. 2014). *Mesembryanthemum crystallinum* L. is a model plant for the study of salt bladders. It has been shown that *Mesembryanthemum crystallinum* L. increases significantly the Na^+^ concentration in its salt bladders after treatment with NaCl (Adams et al. 2005). Treatment of mutants lacking salt bladders with 400 mM NaCl for two weeks resulted in a decrease in Na^+^ and Cl^−^ content within the aboveground tissues and their salt tolerance, demonstrating that salt bladders accumulate excess salt in the body and help to improve its salt tolerance (Agarie et al. 2007). Our results show that the number of salt bladders of *quinoa* increases after treatment with 150 mM NaCl, and that *quinoa* grows better than under normal conditions (Fig. 7A). In addition, we found that the number of PCs of *quinoa* increases under salt stress. These results suggest that salt treatment promotes the development of salt bladders, which in turn enhances salt-secretion and salt-tolerance of *quinoa* and ultimately improves the growth of *quinoa* under salt stress. EBCs on the leaf surface of *Mesembryanthemum crystallinum* L. contribute to plant growth by acting as reservoirs and favor salt tolerance by maintaining ionic segregation and stability within active tissues (Agarie et al. 2007). Similarly, it has been shown that EBCs are also present on the surface of *quinoa*, which increases the seed’s capacity to swell to complete cell division and differentiation under low salt stress (Böhm et al. 2018). These results suggest that the presence of EBCs in *quinoa* increases growth and development and epidermal cell number under salt stress as compared to seedlings grown under control conditions. By subjecting *quinoa* seedlings to low salt treatment and nutrient deprivation, we found that the low concentration of salt may act as a driving force to promote *quinoa* development and salt bladder formation (Fig. 7B-C).

### Nitrogen regulates salt bladder and PC development in *quinoa*

Nitrogen is an essential element in plant growth and development, and nitrogen can increase crop yield and improve crop quality. It has been shown that nitrogen supply strongly affects the relationship between photosynthesis and yield, with higher yields in high-nitrogen than in low-nitrogen grown plants (Bascuñán-Godoy et al. 2018). GO analysis of DEGs in different cell types showed that genes expressed in salt bladders are involved in nitrogen and sulfur metabolism (Fig. 4A). Under sulfur deprivation, the growth of *quinoa* was inhibited. However, the development of salt bladders was significantly promoted (Fig. 8A). Under conditions of nitrogen deficiency, both the growth of *quinoa* leaves and the development of salt bladders were significantly inhibited (Fig. 8A, C). Notably, significant changes in *quinoa* leaf development occurred under nitrogen deprivation, with a significant decrease in the number of salt bladders on the leaf surface and a significant increase in PC size (Fig. 8C). We found that the expression of *ATML1* and *PDF2* homologs, which affect PC development, was significantly reduced, consistent with the results of abnormal PC development (Fig. 8F). Further studies on salt stress treatment of *quinoa* seedlings under nitrogen deprivation showed that although NaCl treatment stimulates the development of salt bladders under these conditions, it did not improve the growth of *quinoa* (Fig. 9A). Nitrogen deficiency is a crucial factor affecting plant development and response to various stresses. During later stages of leaf growth, the density of glandular trichomes may become more nitrogen-limited (Bilkova et al. 2016). This nitrogen limitation can directly impair the production and functionality of these protective structures, weakening the plant’s overall stress response capabilities. In the case of *quinoa*, nitrogen is essential for its growth and the proper development of its distinctive salt bladders. These epidermal structures are instrumental in managing salt stress, and their development is significantly influenced by the levels of nitrogen present in the soil. This study suggests that while salt treatments can initiate the growth of salt bladders, their enhancement is dependent on the presence of sufficient nitrogen (Fig. 8A). Inadequate nitrogen levels may hinder the development of these bladders. Moreover, nitrogen deficiency can impact the expression of genes (Corteggiani Carpinelli et al. 2014) and regulatory pathways that dictate the development of salt bladders. These results suggest that nitrogen is very important for *quinoa* growth and that salt treatment can only enhance the growth of *quinoa* by promoting the development of salt bladder bladders if sufficient nitrogen is available.

## Materials and methods

### Plant material and growth conditions

Plant material was selected from fully matured and healthy *quinoa* seeds. Initially, the seeds were rinsed with water to eliminate impurities, followed by a 3-min soak in a 0.5% sodium hypochlorite solution for sterilization. Subsequently, the seeds were rinsed with distilled water to remove any remaining sodium hypochlorite. The *quinoa* seeds were sown in pots with a diameter of 15 cm, each filled with vermiculite. Ten seeds were planted per pot, and after germination, four uniformly growing plants were retained per pot, with aberrantly small or large plants being removed. The incubation conditions were maintained at 25°C with a photoperiod of 16 h of light and 8 h of darkness. Each treatment consisted of at least four pots, with four plants per pot. The control group was irrigated with 1/2 MS medium, while the salt treatment group received 1/2 MS medium supplemented with 150 mM NaCl. The nitrogen deprivation group was watered with 1/2 MS medium devoid of nitrogen, and the sulfur deficiency group was watered with 1/2 MS medium lacking sulfur. Each experiment was replicated three times to ensure accuracy and reliability.

### cDNA synthesis and RT-qPCR to measure relative gene expression

RNA was extracted from 0.5 mg of *quinoa* leaves using FastPure Plant Total RNA Isolation Kit (Vazyme, China) 0.1 μg of RNA was taken and synthesized using a 4 × gDNA wiper in the HiScript II 1st Strand cDNA Synthesis Kit (Vazyme) before the reverse transcription step to remove genomic contamination. Anchored Oligo (dT) 23 VN was designed for binding site anchoring to synthesize full-length cDNA for cloning and performing RT-qPCR (Reverse transcription quantitative PCR). Specific primers were designed based on the cDNA sequence information of *quinoa* in the GenBank nucleic acid sequence database (Table S10). RT-qPCR was performed on a qTower real-time PCR System (Analytik Jena, Germany) and the enzyme reagent was NovoStart®SYBR qPCR SuperMix plus (novoprotein, China). The PCR system was configured and run according to the instructions provided by the manufacturer, and relative gene expression was calculated using the 2-ΔΔct method.

### Preparation of protoplasts

The true leaves of two-week-old *quinoa* seedlings were cut into strips that were 1–2 mm in length with a razor blade and immersed in a freshly prepared enzymatic solution (refer to the system in the pre-publication article from our lab (Liu et al. 2020)) and subjected to a vacuum for 10 min. The material was placed in the dark and shaken at 40 rpm on a thermostatic shaker at 28°C for 2 h. Isolated protoplasts were washed three times using 8% mannitol buffer and then filtered through a 40 μm cell sieve. The activity of the cells was detected using Taipan blue staining and the cell concentration was counted with a hematocrit plate.

### cDNA library construction and sequencing

The protoplast suspension concentration was adjusted to 1000 / µL and GEMs were generated according to the 10 × Chromium Single-Cell 3 ‘GEM (Gel Beads in-Emulsion) Library & Gel Bead Kit v3.1 instructions. GEMs were conducted with reverse transcription for 45 min at 53°C and 5 min at 85°C to extract cDNA with a 10 × barcode and UMI information. High-quality cDNA libraries were chosen for high-throughput sequencing on the Illumina Nova 6000 Pe15 platform after the cDNA was amplified and examined.

### Single-cell transcriptome data analysis

Raw reads were compared to the *quinoa* reference genome using Cell Ranger 3.0.0, the official software of 10 × genomics, to obtain quality control results such as high-quality cell counts, gene counts, and genome comparison rates of the raw data. The initial quality control results from Cell Ranger analyses were rigorously re-evaluated using the Seurat R package to meticulously exclude data associated with multiple cells, doublets, or unbound cells. Theoretically, the number of genes (nGene), the number of UMIs (nUMI), and the proportion of mitochondrial genes (percent_mito) expressed in most of the cells will be concentrated in a certain region, and based on their distribution characteristics a distribution model can be fitted, which can be used to find out-of-domain values in them and to exclude abnormal data. The quality control criterion was to retain cells with gene counts and UMI counts within ± 2 times the standard deviation of the mean and with mitochondrial gene ratios below 100% as high-quality cells for downstream analysis. Based on the dimensionality reduction results of PCA (Principal Components Analysis), cell cluster clustering was visualized by UMAP (Uniform Manifold Approximation and Projection), and the clustering algorithm SNN (Shared nearest neighbor). The differences between the specified cell clusters and all the remaining cell clusters were tested using the bimodal test to filter the specific marker genes for each cell cluster. The differential gene expression was determined by Wilcoxon rank-sum test available in Seurat, alongside the Benjamini–Hochberg method to correct for multiple testing. Genes with an adjusted p-value below the threshold of 0.05 were identified as marker genes. These markers were subsequently sorted by their log-fold change in expression specific to the cluster of interest, as opposed to their expression in other clusters. DEGs were identified by applying stringent criteria: a p-value threshold of less than 0.05 and a fold-change magnitude of at least 2.

### Pseudotime analysis

Using the monocle 2 R package (Qiu et al. 2017), we screened out the genes with a large degree of variation in gene expression between cells performed spatial dimensionality reduction based on their expression profiles, and constructed a minimum spanning tree (MST). According to the MST branches, the longest paths were found to represent the differentiation trajectories of cells with similar transcriptional profiles. Firstly, extract the data from the Seurat object and create the CellDataSet(CDS) using the newCellDataSet function. The size factor and dispersion were assessed by estimateSizeFactors and estimateDispersions, which helped us standardize mRNA differences between cells and conduct subsequent difference analysis. The genes were filtered by setting the parameters min_expr = 0.1 and num_cells_expressed ≥ 10 to obtain the genes needed for subsequent operations. Seurat was used to select highly variable genes, and setOrderingFilter was used to embed genes into CDS object. The reduceDimension (CDS, max_components 2, method ‘DDRTree’) was used to reduce the dimension of the data.

### Drawing of TF network diagram

To identify differentially expressed TFs specifically enriched in pre-stalk cells (pre-SC), stalk cells (SC), and epidermal bladder cells (EBC), we conducted a comprehensive screening analysis. We performed a protein–protein interaction (PPI) network analysis using the STRING database (version 11.0, RRID: SCR_005223). The outcome of the PPI analysis was downloaded from the STRING database in tab-separated values (TSV) format. Finally, the visualization of the protein–protein interaction network was achieved using Cytoscape (version 3.4.0, RRID: SCR_003032).

## Supplementary Information

Additional file 1: Fig. S1. Observation of plasmodesmata between initial bladder cells and neighboring epidermal cells by transmission electron microscopy. Fig. S2. Observation and analysis of the ultrastructure of mature salt bladders and adjacent epidermal cells. Fig. S3. Observation and analysis of the ultrastructure of mature stalk cells. Fig. S4. Top 57 GO terms of all clusters and expression features of *L0C110682269*. Fig. S5. New marker genes of MMC, pre-SC, SC, and EBC for feature map presentation.

Additional file 2: Table S1. Sequencing summary statistics showing the total number of cells before and after trimming and quality filtering. Table S2. All differentially expressed genes in each cell cluster. Table S3. Top 10 differentially expressed genes in each cell cluster. Table S4. The GO analysis of all differentially expressed genes in each cell cluster. Table S5. The KEGG analysis of all differentially expressed genes in each cell cluster. Table S6. GO enrichment analysis of the genes with highly variable expression of the three identified gene modules. Table S7. GO enrichment analysis of the genes with highly variable expression of the five identified gene modules. Table S8. Differently expressed TFs in each cluster. Table S9. String protein–protein interaction network analysis data. Table S10. The primers for RT-qPCR.

## Data Availability

The raw sequence data reported in this paper have been deposited in the Genome Sequence Archive (Genomics, Proteomics & Bioinformatics 2021) in National Genomics Data Center (Nucleic Acids Res 2022), China National Center for Bioinformation / Beijing Institute of Genomics, Chinese Academy of Sciences (GSA: CRA016880) that are publicly accessible at https://ngdc.cncb.ac.cn/gsa.

## Abbreviations

DEGs: Differentially expressed genes
EBC: Epidermal bladder cells
GC: Guard cell
GO: Gene Ontology
KEGG: Kyoto Encyclopedia of Genes and Genomes
LN: Low nitrogen
LS: Low sulfur
MST: Minimum spanning tree
PCA: Principal Components Analysis
pre-SC: pre-stalk cells
RNA-seq: RNA sequencing
ROS: Reactive oxygen species
RT-qPCR: Reverse transcription quantitative PCR
SC: Stalk cells
scRNA-seq: single cell RNA sequencing
SEM: Scanning electron microscopy
SNN: Shared nearest neighbor
TEM: Transmission electron microscopy
TF: Transcription factor
UMAP: Uniform Manifold Approximation and Projection
UMI: Unique molecular identifiers
UV: Ultraviolet
