# Supplementary material for: Single-cell transcriptomic analysis reveals the developmental trajectory and transcriptional regulatory networks of quinoa salt bladders
Source: Stress Biol. 2024 Nov 13;4(1):47. doi: 10.1007/s44154-024-00189-3 (PMC11557854; doi:10.1007/s44154-024-00189-3)
Supplement: Supplementary file 1 — Additional file 1: Fig. S1. Observation of plasmodesmata between initial bladder cells and neighboring epidermal cells by transmission electron microscopy. Fig. S2. Observation and analysis of the ultrastructure of mature salt bladders and adjacent epidermal cells. Fig. S3. Observation and analysis of the ultrastructure of mature stalk cells. Fig. S4. Top 57 GO terms of all clusters and expression features of L0C110682269. Fig. S5. New marker genes of MMC, pre-SC, SC, and EBC for feature map presentation. [file 44154_2024_189_MOESM1_ESM.docx]

Supplementary figure


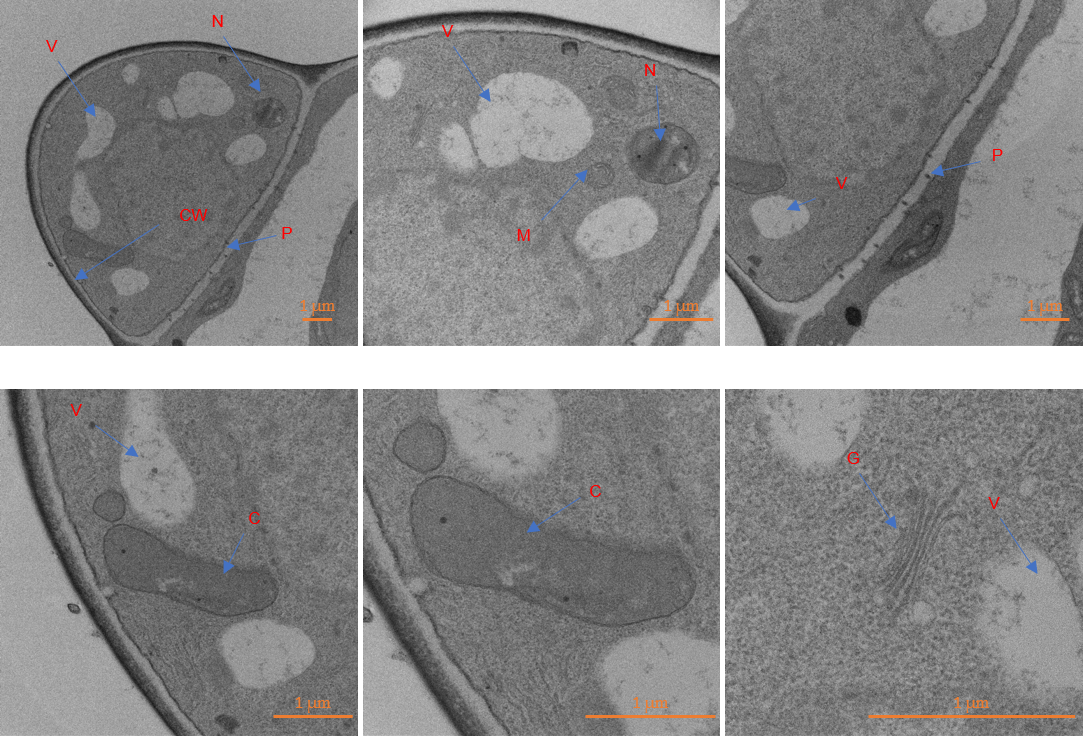


**Fig. S1** Observation of intercellular connecting filaments between initial bladder cells and neighboring epidermal cells by transmission electron microscopy.

The figure displays a micrograph of the ultrastructure within the progenitor vesicle cells. CW: cell wall, V: vacuole, N: nuclei, M: mitochondria, C: chloroplasts, G: Golgi apparatus, P: plasmodesmata. Scale bar: 1 μm.


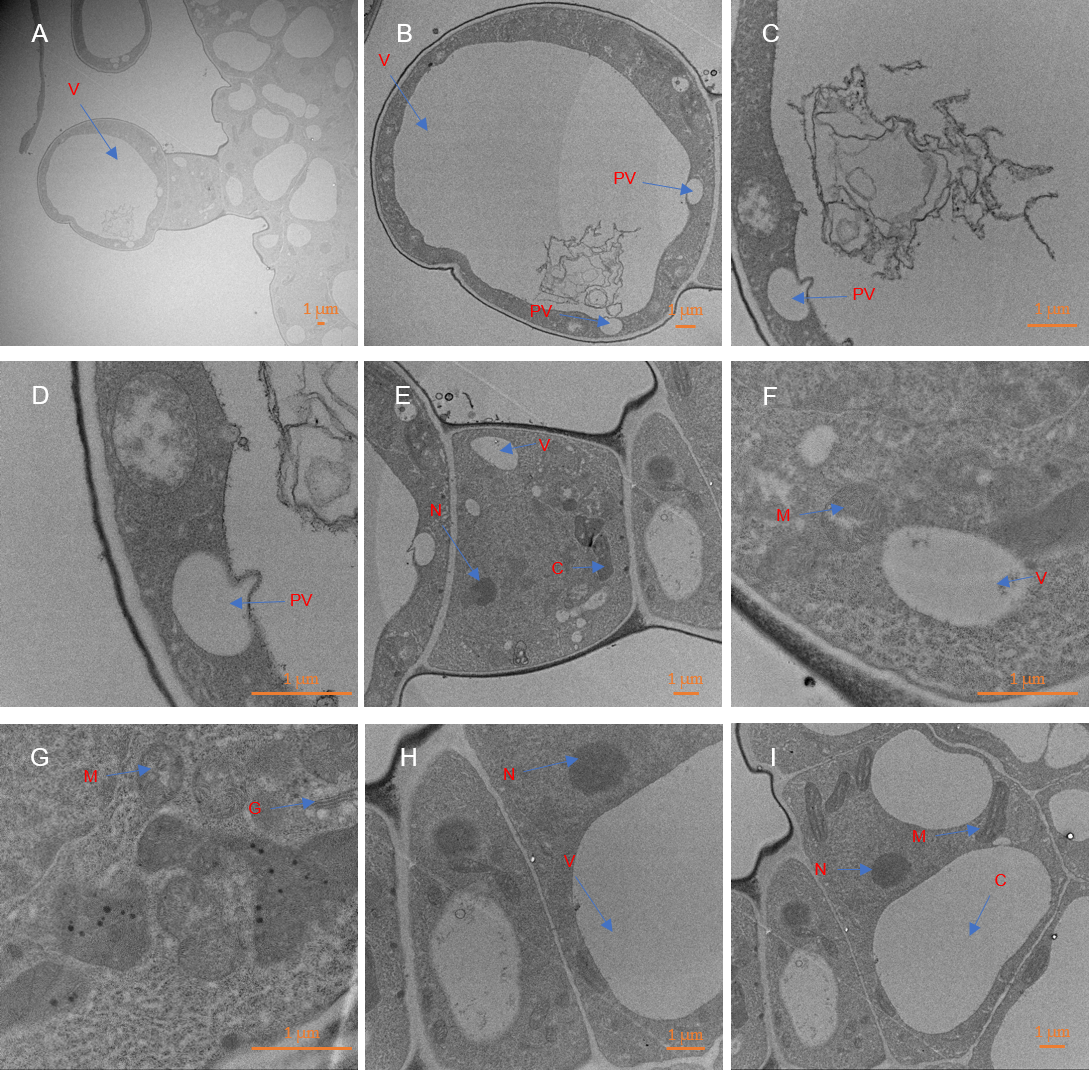


**Fig. S2** The observation and analysis of the ultrastructure of mature salt bladders and adjacent epidermal cells.

**A-D** Characteristic distribution of large vacuoles, Golgi apparatus, and autophagic vacuoles in mature EBCs. **E-G** Characteristic distribution of nuclei, mitochondria, chloroplasts, and Golgi apparatus in stalk cells. **H-I** Characteristic distribution of organelles in the epidermal cells adjacent to the salt bladder. V: vacuole, N: nuclei, M: mitochondria, C: chloroplasts, G: Golgi apparatus, PV: phagocytic vacuole. Scale bar: 1 μm.


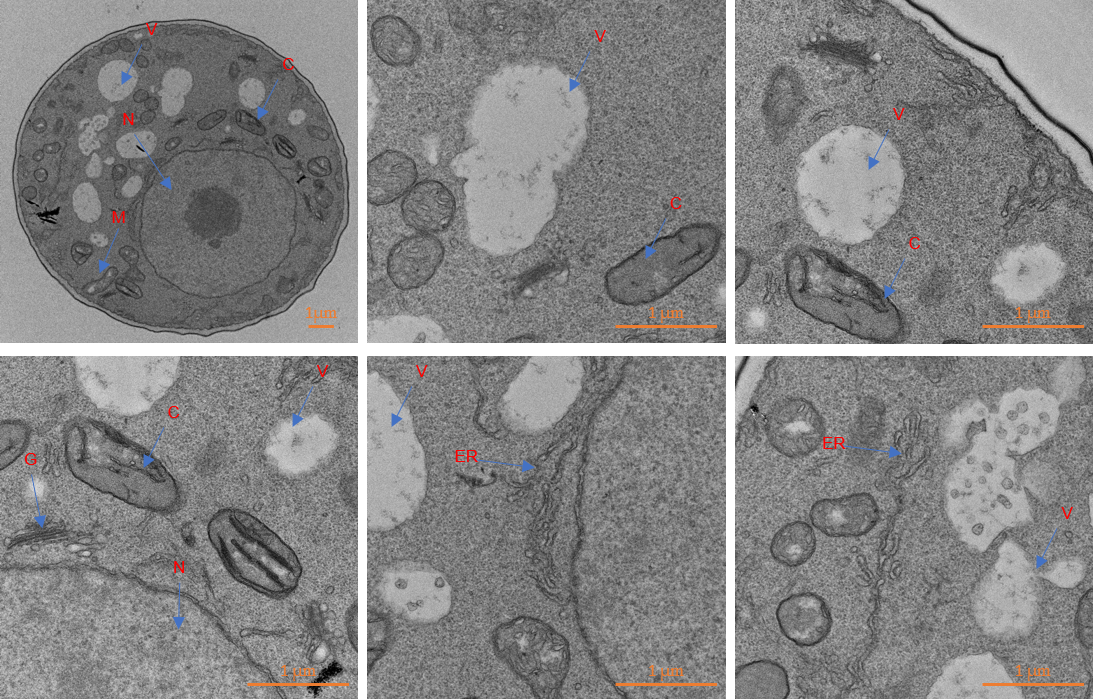


**Fig. S3** Observation and analysis of the ultrastructure of mature stalk cells.

Images of the ultrastructure of various organelles within mature stalk cells. V: vacuole, N: nuclei, M: mitochondria, C: chloroplasts, G: Golgi apparatus, ER: endoplasmic reticulum. Scale bar: 1 μm.


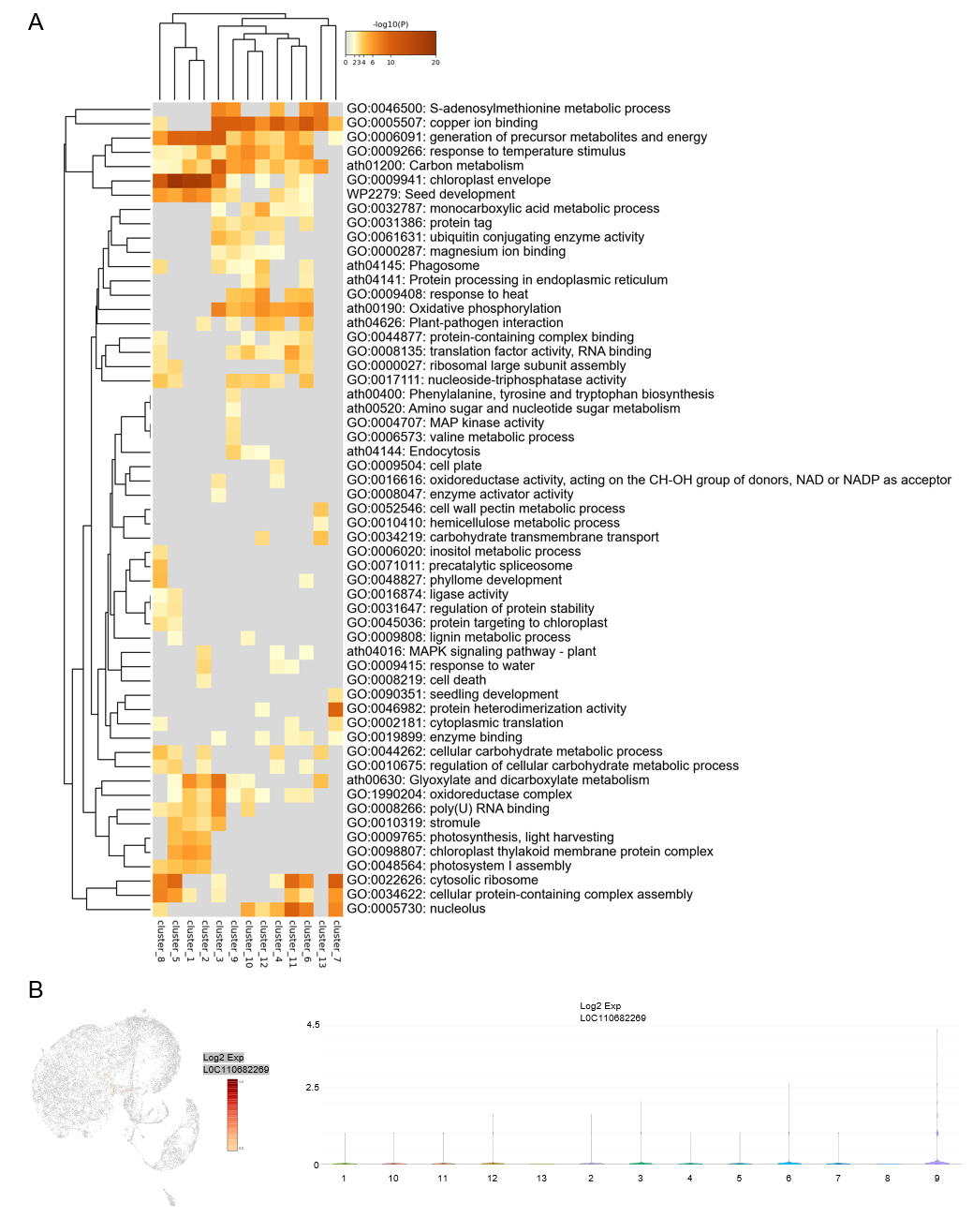


**Fig. S4** The top 57 GO terms of all clusters and expression feature of *L0C110682269*.

(A) GO enrichment analysis of DEGs in 13 cell clusters. (B) Feature maps and violin maps show the characteristics of gene expression of *L0C110682269*.


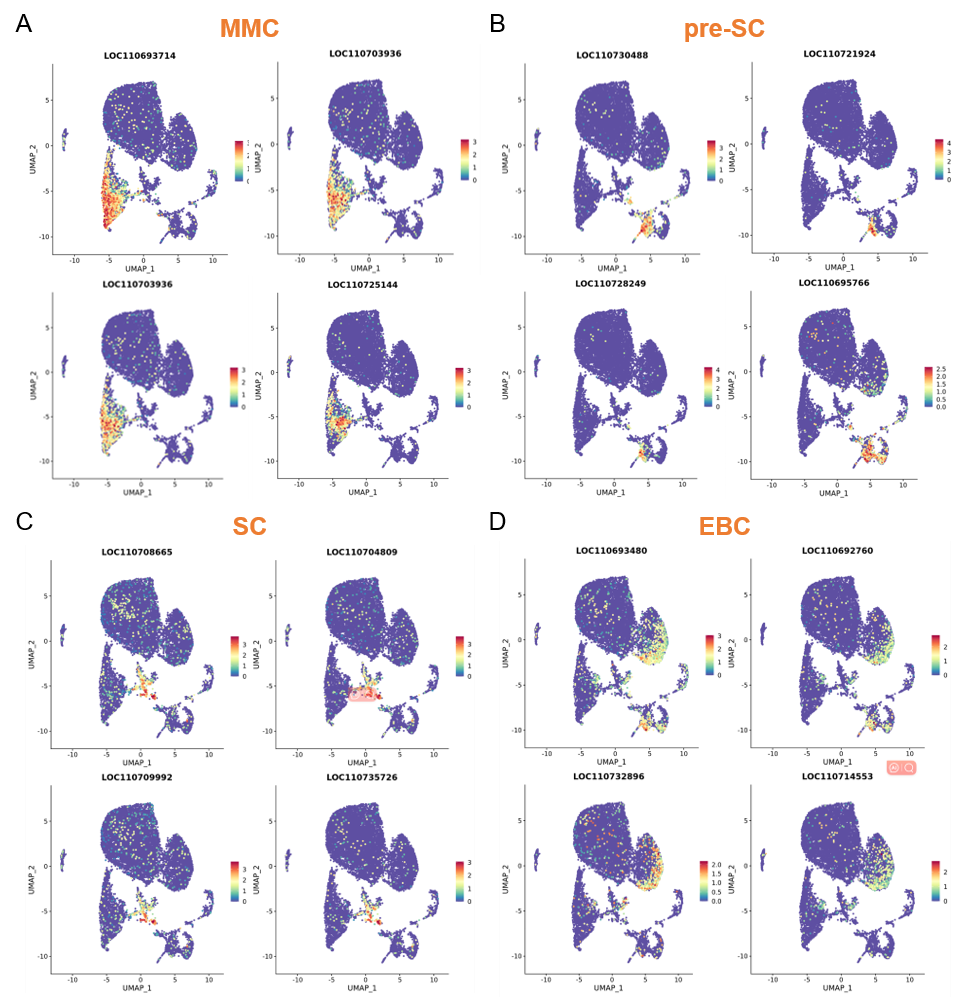


**Fig. S5** The new marker genes of MMC, pre-SC, SC, and EBC for feature map presentation.
